# Synthesis and radioprotective effects of novel hybrid compounds containing edaravone analogue and 3‐*n*‐butylphthalide ring‐opening derivatives

**DOI:** 10.1111/jcmm.16557

**Published:** 2021-05-08

**Authors:** Xuejiao Li, Xinxin Wang, Longfei Miao, Yahong Liu, Xiaona Lin, Yuying Guo, Renbin Yuan, Hongqi Tian

**Affiliations:** ^1^ Tianjin Key Laboratory of Radiation Medicine and Molecular Nuclear Medicine Institute of Radiation Medicine Peking Union Medical College and Chinese Academy of Medical Science Tianjin China

**Keywords:** 3‐*n*‐butylphthalide, edaravone, ionizing radiation, radioprotector, reactive oxygen species

## Abstract

As the potential risk of radiation exposure is increasing, radioprotectors studies are gaining importance. In this study, novel hybrid compounds containing edaravone analogue and 3‐*n*‐butylphthalide ring‐opening derivatives were synthesized, and their radioprotective effects were evaluated. Among these, compound **10a** displayed the highest radioprotective activity in IEC‐6 and HFL‐1 cells. Its oral administration increased the survival rates of irradiated mice and alleviated total body irradiation (TBI)‐induced hematopoietic damage by mitigating myelosuppression and improving hematopoietic stem/progenitor cell frequencies. Furthermore, **10a** treatment prevented abdominal irradiation (ABI)‐induced structural damage to the small intestine. Experiment results demonstrated that **10a** increased the number of Lgr5^+^ intestinal stem cells, lysozyme^+^ Paneth cells and Ki67^+^ transient amplifying cells, and reduced apoptosis of the intestinal epithelium cells in irradiated mice. Moreover, in vitro and in vivo studies demonstrated that the radioprotective activity of **10a** is associated to the reduction of oxidative stress and the inhibition of DNA damage. Furthermore, compound **10a** downregulated the expressions of p53, Bax, caspase‐9 and caspase‐3, and upregulated the expression of Bcl‐2, suggesting that it could prevent irradiation‐induced intestinal damage through the p53‐dependent apoptotic pathway. Collectively, these findings demonstrate that **10a** is beneficial for the prevention of radiation damage and has the potential to be a radioprotector.

## 1 INTRODUCTION

With the increasing use of nuclear technology, the potential risk of radiation exposure is becoming a big cause of concern.^1^ People are prone to ionizing radiation (IR) exposure during terror attacks, nuclear accidents, space exploration and radiotherapy.^2^, ^3^, ^4^ IR could cause destructive effect on normal cells in two ways: radiation energy acts directly on biological macromolecules and leads to ionisation or excitation of macromolecules, this is the direct effect of IR; rays act on water molecules and accelerate the generation of reactive oxygen species (ROS), which may attack the surrounding macromolecules, this is the indirect effect of IR.^5^ As a result, IR can impair the structure of DNA, proteins and lipids, and eventually lead to deleterious consequences, including cell apoptosis and organ system dysfunction.^6^, ^7^, ^8^ Research has shown that hematopoietic tissues and the gastrointestinal (GI) tract are sensitive to IR, the former being the most sensitive,^9^, ^10^ and there are few effective methods to prevent or mitigate radiation‐induced injury of the GI tract.^11^, ^12^ Thus, finding remedies to protect these systems from radiation damage is of utmost importance.^13^, ^14^

Radioprotectors are compounds used before or at the time of radiation exposure to inhibit radiation damage and protect biomacromolecules.^5^ To date, numerous natural products and synthetic compounds have been evaluated for their potential as effective radioprotectors.^3^, ^15^ Among these, amifostine is the only radioprotective agent approved by the Food and Drug Administration (FDA) for decreasing adverse reactions in patients undergoing radiation therapy.^4^ However, amifostine can only be administrated intravenously and has been shown to have multiple adverse effects, such as nausea, hypotension, cephalalgia, fainting and transient hypocalcemia.^4^, ^5^, ^13^ Hence, there is an urgent need to develop efficient, convenient and less toxic radioprotectors to protect normal human tissues from radiation damage.

**FIGURE 1:**
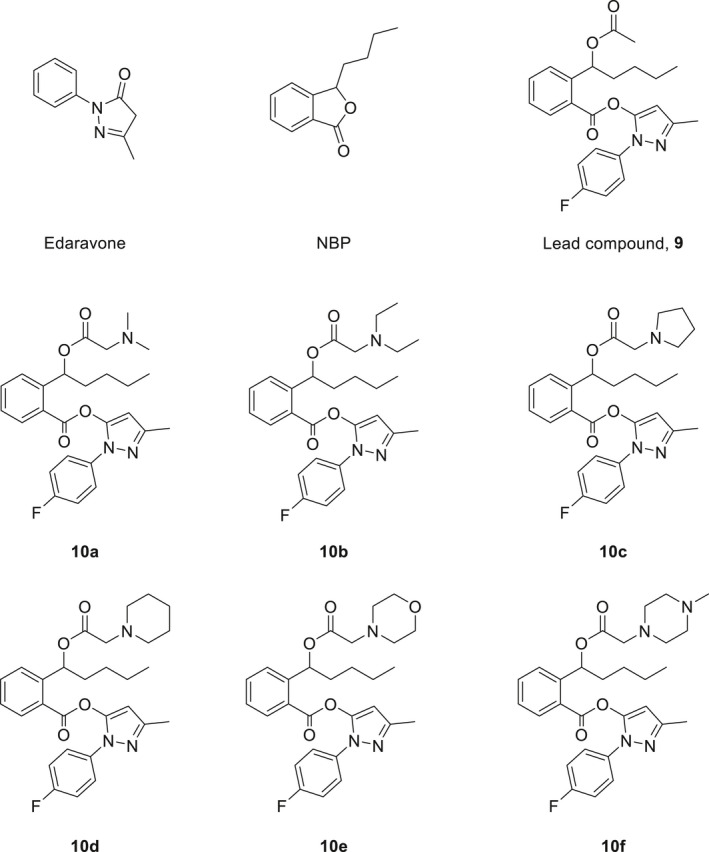
Structures of edaravone, 3‐*n*‐butylphthalide, the lead compound and the target compounds

Edaravone (3‐methyl‐1‐phenyl‐2‐pyrazolin‐5‐one, Figure 1), generally known as an effective free radical scavenger with low toxicity, was approved by the Japanese Ministry of Health in 2001 for the treatment of ischemic stroke.^16^ Recent evidence has indicated that edaravone could significantly increase the survival rate of mice exposed to whole‐body X‐ray irradiation and protect human peripheral blood lymphocytes against γ‐ray‐induced radiation damage.^17^ The bioactivity of edaravone is due to the removal of toxic ROS and the inhibition of DNA damage caused by oxidative stress.^18^, ^19^ Unfortunately, its oral use is limited owing to its poor aqueous solubility and low intestinal permeability.^20^ The 3‐*n*‐butylphthalide (NBP, Figure 1), as a native compound, was approved by the State Food and Drug Administration (SFDA) of China for the therapy of ischemic stroke in 2002.^21^, ^22^ Multiple studies have shown that NBP inhibits platelet aggregation and thrombus formation, promotes cerebral microcirculation and decreases oxidative damage.^23^, ^24^, ^25^ In addition, toxicology studies have demonstrated safety of NBP as a drug.^26^ However, its potency is restricted owing to its poor aqueous solubility and low bioavailability.^23^, ^24^ In a previous study, Sheng *et al* designed and synthesized a range of hybrid compounds containing edaravone analogues and NBP derivative, one of which exhibited potent anti‐ischemic stroke activity via the synergistic action of edaravone analogue and NBP, and could be administrated orally to rats.^27^, ^28^ They also proved that the mechanisms underlying its bioactivity include inhibiting platelet aggregation, scavenging free radicals and reducing oxidative stress.^27^, ^28^

Since the biological activities of the compounds containing edaravone analogue and NBP ring‐opening derivative are related to their free radical scavenging effects, our study aimed to investigate whether these compounds exert radioprotective activities. We used the hybrid compound identified by Sheng *et al* as the lead compound, and synthesized a group of its derivatives (Figure 1). We hypothesized that these compounds could exhibit potent radioprotective activities via the synergistic action of the edaravone analogue and NBP. To enhance the aqueous solubility and oral bioavailability of the lead compound, we modified its structure by introducing various amines, for example, dimethylamine, pyrrolidine and morpholine, to the side chain of the ring‐opening NBP. After obtaining a series of novel hybrid compounds, we examined their in vitro and in vivo radioprotective effects and mechanisms. Our findings provide a new direction for studies on radioprotectors.

## 2 MATERIALS AND METHODS

### 2.1 Synthesis and characterization of the compounds

The synthesis of compound **9** and target compounds **10a‐10f** are shown in Scheme S1. All intermediates and compound **9** were synthesized as previously described.^21^, ^23^, ^28^ The synthesis of compounds **10a‐10f** are presented in the Supporting Information. All compounds were characterized using ^1^H NMR and MS, the target compounds **10a‐10f** were also characterized using ^13^C NMR and HRMS (Figure S3‐S32).

### 2.2 Cell culture and irradiation

Rat intestinal epithelial cell 6 (IEC‐6) and human fetal lung fibroblast 1 (HFL‐1) were used for in vitro studies. IEC‐6 and HFL‐1 cells were cultured in DMEM and Ham's F12K medium, respectively, supplemented with 10% fetal bovine serum and penicillin (100 U/mL)/streptomycin (100 μg/mL), in a humidified atmosphere with 5% CO_2_ at 37°C. A ^137^Cs γ‐ray source placed in an Exposure Instrument Gammacell‐40 (Atomic Energy of Canada Ltd.) was used for in vitro experiments at a dose rate of 0.98 Gy/min.

### 2.3 Cell viability assay

For the cytotoxicity experiment, IEC‐6 and HFL‐1 cells were plated in 96**‐**well plates (5 × 10^3^ cells per well), incubated overnight, and treated with the tested compounds at concentrations from 0 to 100 μmol/L. After 24 hours, the cells were washed once, and cell viability was measured using the luminescence‐based CellTiter Glo TM assay according to the manufacturer's protocols (Promega).

To evaluate the radioprotective effects, IEC‐6 and HFL‐1 cells were plated in 96**‐**well plates (5 × 10^3^ cells per well) and incubated overnight. The cells were pretreated with the tested compounds, with edaravone as the positive control. After 1 hour, the cells were irradiated with 8.0 or 10.0 Gy and were cultured for 24 hours. Cell viability was measured as described above.

### 2.4 Measurement of intracellular ROS levels in IEC‐6 cells

IEC‐6 cells were plated in 6**‐**well plates (3 × 10^5^ cells per well) and incubated overnight. The cells were pretreated with **10a** for 1 hour, irradiated with 4.0 Gy, and harvested after 1 hour. Then, they were incubated with 2’,7’‐dichlorodihydrofluorescein diacetate (DCFDA; 10 μmol/L) for 20 minutes at 37°C. The levels of intracellular ROS were measured using the mean fluorescence intensity (MFI) of DCFDA through flow cytometry (BD Biosciences).

### 2.5 Alkaline single‐cell gel electrophoresis (comet assay)

IEC‐6 cells were plated in 6**‐**well plates at 2 × 10^5^ cells per well, incubated overnight, and then pretreated with **10a** for 1 hour before 4.0 Gy γ‐irradiation. The cells were harvested and suspended in cold PBS. The cell suspension was mixed with 70% low‐melting point agarose (7.5 g/L), and 50 μL of the mixture was placed on a comet slide coated with one layer of normal‐melting point agarose (7.5 g/L). The slides were submerged in precooled lysis buffer (2.5 mol/L NaCl, 100 mmol/L Na_2_EDTA, 10 mmol/L Tris‐HCl, pH 10, 10% DMSO, and 1% Triton X‐100) at 4°C for 2.5 hours and in cold electrophoresis buffer (300 mmol/L NaOH, 1 mmol/L Na_2_EDTA, pH >13) at 4°C for 20 minutes. Electrophoresis was performed at 30 V for 20 minutes. The slides were neutralized with Tris‐HCl (pH 7.5) for 30 minutes and stained with ethidium bromide (50 μL per slide, 2.5 μg/mL). Finally, the slides were observed using a fluorescence microscope (Olympus). The percentage of DNA in the tail and Olive tail moment were measured using the Comet Assay Software Project.

### 2.6 Analysis of nuclear factor erythroid 2‐related factor 2 (Nrf2) expression by immunofluorescence

IEC‐6 cells were cultured in 12‐well plates with chamber slides (5 × 10^4^ cells per well) and incubated overnight. After treated with **10a** for 1 hour, the cells were irradiated with 6.0 Gy and cultured for another 24 hours. The cells were fixed with 4% paraformaldehyde for 30 minutes and then permeabilized in Triton X‐100 for 15 minutes. After blocked with 5% bovine serum albumin (BSA) for 3 hours, the slides were incubated with anti‐Nrf2 antibody (1:200 dilution, Proteintech) overnight at 4℃ and stained with FITC‐conjugated secondary antibody (1:50 dilution, Proteintech) for 1 hour in dark. The cell nuclei were stained with DAPI‐containing sealing agent and images were taken using a fluorescence microscope (Life Technologies).

### 2.7 Protein extraction and western blotting

IEC‐6 cells were plated in 6**‐**well plates with 5 × 10^5^ cells per well, incubated overnight and pretreated with **10a** for 1 hour before 6.0 Gy of γ‐irradiation. After 24 hours of incubation, the cells were collected. To extract the total proteins, the cells were lysed with cold RIPA buffer supplemented with protease inhibitor cocktail and phenylmethylsulfonyl fluoride. And a Nuclear/Cytoplasmic Protein Extraction kit was used to extract the nuclear proteins. Equal amounts of proteins were resolved on a 10% SDS–PAGE gel, and the proteins were electroblotted onto polyvinylidene difluoride membranes. The membranes were blocked with TBST buffer containing 5% BSA and incubated for 1.5 hours at room temperature with antibodies recognizing Nrf2 (1:1000 dilution, Proteintech), NAD(P)H quinone oxidoreductase 1 (NQO1, 1:20 000 dilution, Abcam), heme oxygenase 1 (HO‐1, 1:3000 dilution, Proteintech), Bax (1:5000 dilution, Abcam), Bcl‐2 (1:1000 dilution, Abcam), Lamin B1 (1:5000 dilution, Proteintech) and GAPDH (1:20 000 dilution, Proteintech). The membranes were then incubated with the corresponding secondary antibodies for 1 hour. The protein bands were visualized using ECL chemiluminescence reagents, and their intensities were analysed using Image Lab™ software (Bio‐Rad).

### 2.8 Animal experiments

Male C57BL/6 mice were purchased from Beijing Huafukang Bioscience Co. Inc and raised under pathogen‐free conditions at the experimental animal centre of the Institute of Radiation Medicine (IRM), Chinese Academy of Medical Sciences (CAMS). Mice aged 6‐8 weeks were used in our experiments. All animal experiments in this study were approved by the Institutional Animal Care and Use Committee of the IRM, CAMS.

Irradiation was performed using a ^137^Cs γ‐radiation source at a dose rate of 0.98 Gy/min. For the total body irradiation (TBI) survival experiment, mice were randomly assigned to five groups, namely control, TBI + vehicle, TBI + **10a** (150 mg/kg), TBI + **10a** (300 mg/kg) and TBI + **10a** (600 mg/kg) (n = 10 per group). In addition, mice were randomly assigned to 4 groups in the remaining TBI experiments, namely control, **10a** (300 mg/kg), TBI + vehicle, and TBI + **10a** (300 mg/kg) (n = 5 per group). The mice in the control group and those treated with **10a** alone were sham‐irradiated. For abdominal irradiation (ABI) studies, a lead shield was used to protect other parts of the body. The mice were randomly divided into three groups in the survival experiment (n = 10 per group) and in the remaining ABI experiments (n = 5), namely control, ABI + vehicle, and ABI + **10a** (300 mg/kg). Mice in the control group were sham‐irradiated. In all animal experiments, 15% Solutol HS 15 with 20% HP‐β‐CD in purified water was used to dissolve compound **10a**. The mice of the IR +**10a** groups were treated with gavage‐administered **10a** at the indicated dose 1 hour before radiation, whereas those in the **10a** groups received **10a** at the same dose and through the same route. Mice in the control and IR +vehicle groups were treated with vehicle using the same method as used for the treatment of **10a**.

### 2.9 Survival study

Mice in the irradiation groups received 8.0 Gy TBI or 15.0 Gy ABI. The survival status of each mouse was observed for 30 days after irradiation, and the results were expressed as survival rates.

### 2.10 Peripheral blood cell and bone marrow nucleated cell counts

To assess the hematopoietic injuries, mice in the irradiation groups were exposed to 4.0 Gy of TBI and euthanized after 14 days. Peripheral blood was obtained from the eyes and placed in EP tubes containing K_3_EDTA. White blood cells (WBCs), platelets (PLTs), the percentage of neutrophils (NE%), and the percentage of lymphocytes (LY%) were analysed by hematology analyzer (Nihon Kohden). Bone marrow nucleated cells were flushed out from the mouse femurs and were counted similarly.

### 2.11 Flow cytometry

In this study, LSK (Lineage^‐^Sca1^+^c‐kit^+^) was used to define hematopoietic stem cells (HSCs), and Lineage^‐^Sca1^‐^c‐kit^+^ was used to define hematopoietic progenitor cells (HPCs) as suggested in previous studies.^29^, ^30^ For HPC and LSK analysis, bone marrow cells were flushed out of the femurs and filtered. Bone marrow cells (5 × 10^6^) were incubated with biotin‐labelled antibodies specific for murine CD4, B220, CD11b, Gr1, CD8 and Ter119 (mixed Lineage antibodies, Biolegend) and subsequently stained using PerCP streptavidin (Biolegend), PE sca1 antibody (eBioscience), and APC c‐kit antibody (eBioscience). HPCs and LSKs were detected using flow cytometry and analysed using the BD Accuri C6 software.

### 2.12 Analysis of intracellular ROS levels in HPCs and LSKs

Bone marrow cells (5 × 10^6^) were stained with HPC and LSK antibodies and incubated with DCFDA at the concentration of 10 μmol/L. The levels of intracellular ROS were assessed by measuring the MFI of DCFDA using the flow cytometry.

### 2.13 Histological analysis

To assess the intestinal damage, mice in ABI groups were irradiated with 15.0 Gy, and euthanized on the fifth day after ABI. The small intestinal tissues of the mice were resected, fixed in 10% neutral‐buffered formalin solution, dehydrated and embedded in paraffin. The blocks were cut into 4‐μm‐thick sections, stained with haematoxylin–eosin (H&E), and observed under a microscope (Olympus). For the morphological analysis, six circular transverse sections were analysed and quantitated per mouse to determine the villi height and crypts number.

### 2.14 Immunohistochemistry analysis

Sections (4‐μm thick) were dewaxed and rehydrated in decreasing concentrations of ethanol. Next, antigen retrieval was then performed and the samples were blocked with serum for 1 hour at 37℃. Then, the sections were stained overnight at 4℃ with the following antibodies: anti‐Lgr5 (1:200 dilution, Bioworld), anti‐Lysozyme (1:100 dilution, Bioworld), anti‐Ki67 (1:200 dilution, Abcam), or anti‐Villin (1:100 dilution, Bioworld), followed by incubation with the corresponding secondary antibodies for 40 minutes at 37°C. Subsequently, the cells were stained using DAB kit. The images were captured using a microscope (Olympus), and positive staining was objectively analysed.

### 2.15 TUNEL assay

Sections (4‐μm thick) were treated in accordance with the manufacturer's protocols (Roche), observed using a microscope (Olympus), and analysed using a blind method.

### 2.16 Immunofluorescence analysis

Sections (4‐μm thick) were deparaffinized and rehydrated as described above. Antigen retrieval was performed, and the samples were blocked with serum at 37 ℃. The sections were stained overnight at 4 ℃ with the following antibodies: anti‐γ‐H2AX (1:400 dilution, Bioworld), anti‐p53 (1:100 dilution, Bioworld), anti‐caspase‐3 (1:500 dilution, CST), or anti‐caspase‐9 (1:50 dilution, CST). The sections were stained with the corresponding secondary antibodies in dark. Finally, the sections were washed and counterstained with DAPI. Images were taken using a fluorescence microscope (Olympus).

### 2.17 Statistical analysis

All data are expressed as the mean ± SEM, unless indicated otherwise. The Kaplan–Meier method and log‐rank test were used to analyse the survival rates, whereas unpaired t‐tests (two‐tailed) were used for mean comparisons. Statistical analysis was performed using the GraphPad Prism version 5 software. Differences were considered statistically significant at *P* < 0.05.

## 3 RESULTS

### 3.1 Synthesis of compound 9 and target compounds 10a‐10f

The compounds were synthesized as described in the Supporting Information. Compounds **9** and **10a‐10f** with purities > 95% were used for all the experiments (see Supporting Information) and their oil–water partition coefficient (LogP) values are listed in Table S1. We found that the LogP values of **10a**, **10e**, and **10f** were less than 5, indicating good aqueous solubilities.

### 3.2 Cytotoxicity and radioprotective activity of the tested compounds

**FIGURE 2:**
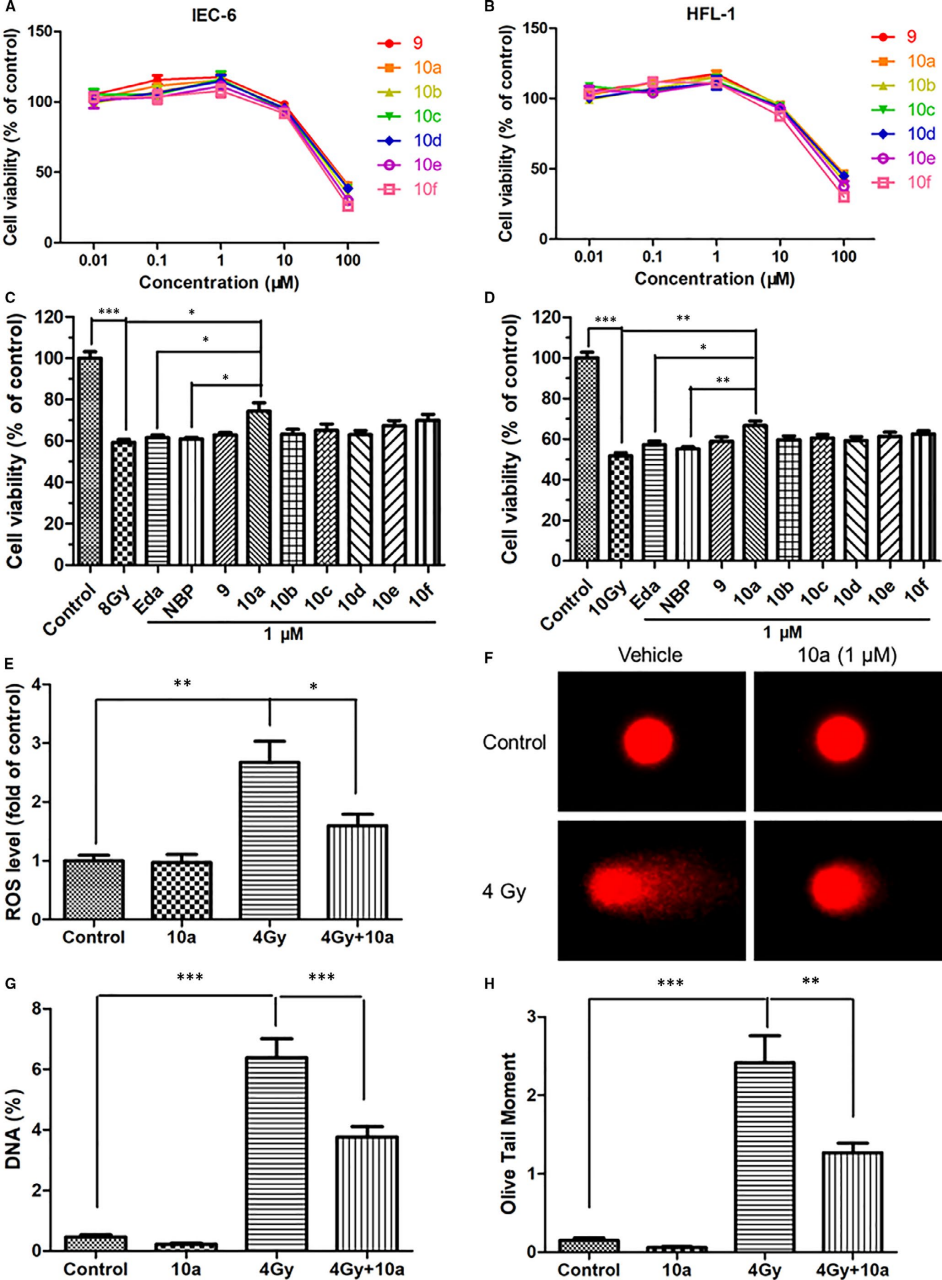
Compound **10a** protected IEC‐6 and HFL‐1 cells from radiation damage. Cell toxicity of the tested compounds on A, IEC‐6 and B, HFL‐1 cells, and radioprotective effects of the tested compounds on C, IEC‐6 and D, HFL‐1 cells are shown. E, Relative ROS levels in irradiated IEC‐6 cells. F, Representative images of comets in IEC‐6 cells. Statistical analyses of G, the percentage of DNA in the tail and H, Olive tail moment based on the comet assay are shown. Data are presented as the mean ± SEM (n = 3). *P < 0.05, **P < 0.01, ***P < 0.001. Eda, edaravone

Considering that intestines and lungs are sensitive to radiation,^31^, ^32^ we used IEC‐6 and HFL‐1 cells to investigate the in vitro radioprotective activities of the tested compounds. Initially, we evaluated the toxicity of compound **9** and target compounds **10a‐10f** on IEC‐6 and HFL‐1 cells. As illustrated in Figure 2A and B, the tested compounds had no toxic effect on IEC‐6 and HFL‐1 cells at lower concentrations and exhibited more pronounced inhibitory effect on the cells at concentrations higher than 10 μmol/L. Thus, a concentration of 1 μmol/L was used for subsequent in vitro experiments.

Then we investigated the radioprotective activity of edaravone, NBP, compound **9** and compounds **10a‐10f** on IEC‐6 and HFL‐1 cells. The radioprotective effects of the tested compounds were displayed in Figure 2C and D. The survival rate of the IEC‐6 cells decreased significantly after 8.0 Gy irradiation, whereas that of the HFL‐1 cells decreased dramatically after 10.0 Gy irradiation (*P* < 0.001). For the treatment groups, the cell survival rates were increased compared with the cells treated with irradiation only. In particular, **10a** remarkably increased the survival rates of IEC‐6 and HFL‐1 cells (*P* < 0.05), and the survival rates of **10a**‐treated cells were higher than those of edaravone‐treated cells (*P* < 0.05), indicating that **10a** has the highest radioprotective activity among the tested compounds.

### 3.3 Compound 10a inhibited IR‐induced oxidative stress in IEC‐6 cells

Excessive ROS levels triggered by irradiation may lead to oxidative stress, contributing to cell and tissue damage. As demonstrated in Figure 2E, the ROS level increased significantly in the IR group compared with that in the control group (*P* < 0.01), and that in the **10a** treatment group was only 60% of the IR group (*P* < 0.05). These results suggested that **10a** can scavenge ROS and reduce oxidative stress in irradiated IEC‐6 cells.

### 3.4 Compound 10a protected IEC‐6 cells from IR‐induced DNA damage

Nuclear DNA is an intracellular target of radiation injury. Both direct deposition of radiant energy and excessive oxidative stress in cells induced by rays would aggravate the accumulation of DNA damage.^29^ Figure 2F displays the undamaged and damaged DNA under different treating conditions visualized using a comet assay. The non‐irradiated cells did not show any DNA trailing, whereas irradiated cells showed marked DNA trailing. Further, DNA trailing decreased in the cells of the **10a** pre‐treatment group compared with those of the IR group. We observed an increase in the percentage of DNA in the tail and Olive tail moment after 4.0 Gy irradiation (*P* < 0.001, Figure 2G, H). Compared with the IR group, the percentage of DNA in the tail and Olive tail moment were dramatically reduced upon **10a** pretreatment (*P* < 0.01). These data demonstrated that **10a** played a crucial role in protecting DNA from radiation damage.

### 3.5 Compound 10a upregulated the expression of Nrf2 and downstream antioxidant proteins in irradiated IEC‐6 cells

**FIGURE 3:**
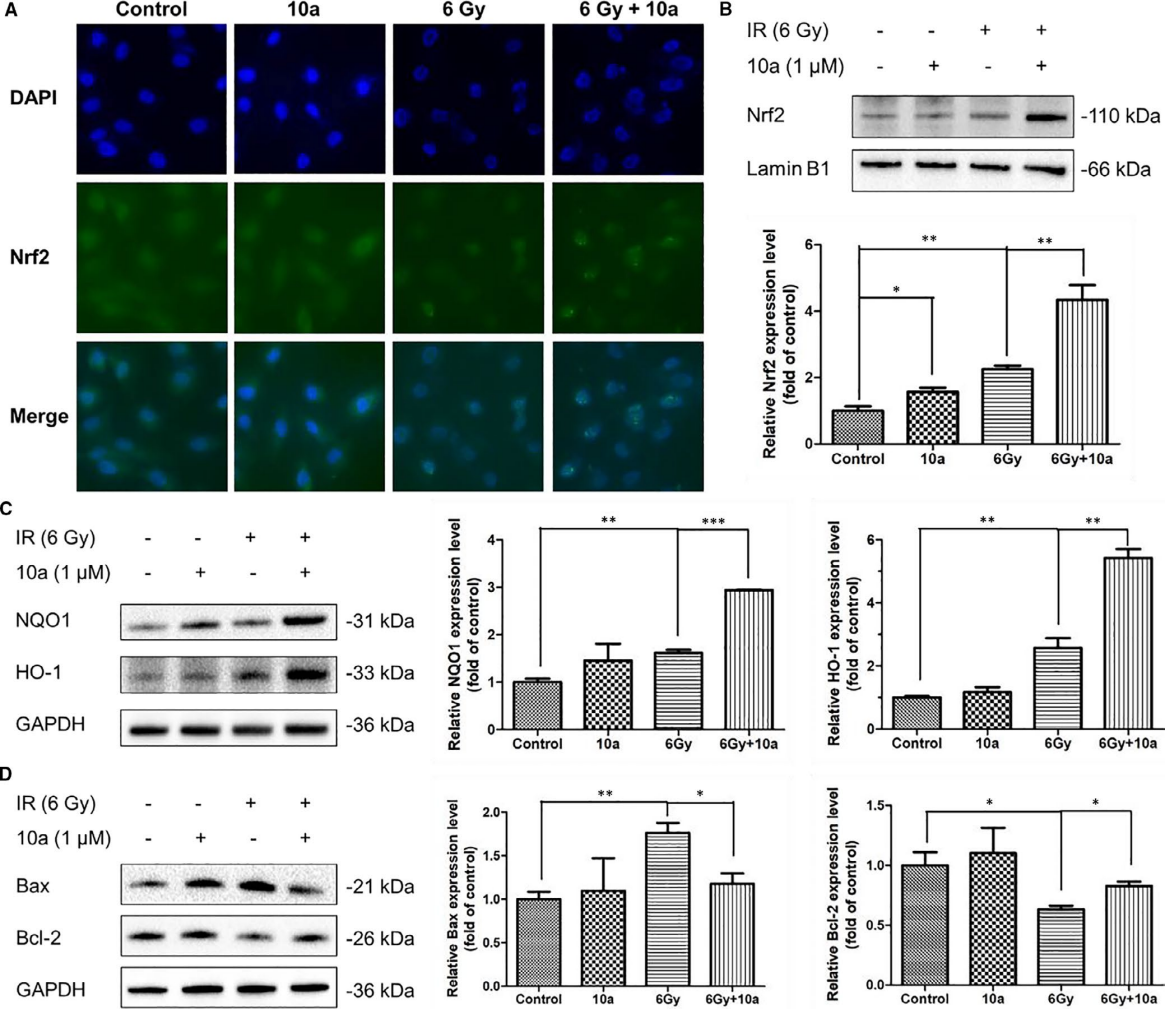
Compound **10a** regulated the expression of Nrf2, its target proteins, and apoptosis‐related proteins in irradiated IEC‐6 cells. A, Representative images showing the expression of Nrf2 in IEC‐6 cells (green, Nrf2; blue, DAPI). B, Western blot analysis of Nrf2 in the nucleus of IEC‐6 cells. Relative quantification of the expression of Nrf2 is shown. C, Western blot analysis of NQO1 and HO‐1 in IEC‐6 cells. Relative quantifications of the NQO1 and HO‐1 proteins are shown. D, Western blot analysis of Bax and Bcl‐2 in IEC‐6 cells. Relative quantifications of the Bax and Bcl‐2 proteins are shown. Data are presented as the mean ± SEM (n = 3). *P < 0.05, **P < 0.01, ***P < 0.001

Nrf2 is a transcription factor which can regulate redox homeostasis in cells.^33^ When activated under stress, it translocates from the cytoplasm to the nucleus and induces the transcription of downstream cytoprotective proteins.^33^, ^34^ Therefore, we analysed the distribution of Nrf2 in IEC‐6 cells by immunofluorescence assay. As displayed in Figure 3A, exposure to IR caused Nrf2 foci formation in the nucleus of the cells, which was enhanced by **10a** treatment. Furthermore, we investigated the expression of Nrf2 in the nucleus of IEC‐6 cells by Western blotting. As shown in Figure 3B, the expression of Nrf2 was upregulated by irradiation, and compound **10a** further increased its expression level. Together, these results suggested that **10a** could induce the nuclear translocation of Nrf2 in irradiated cells.

NQO1 and HO‐1 are enzymes which can be upregulated by Nrf2, and they are known to protect against oxidative injury.^34^, ^35^ We evaluated the levels of antioxidant enzymes NQO1 and HO‐1 in IEC‐6 cells using Western blotting. As illustrated in Figure 3C, the levels of these compounds in IEC‐6 cells significantly increased after irradiation (*P* < 0.01), and **10a** treatment further enhanced their expressions compared to the IR group (*P* < 0.01). These results suggested that **10a** may reduce oxidative stress injury in irradiated cells via the activation of the Nrf2 pathway.

### 3.6 Compound 10a protected IEC‐6 cells against irradiation partially through the modulation of the apoptotic pathway

To further investigate the radioprotective mechanisms of **10a**, we analysed the levels of the pro‐apoptotic protein Bax and anti‐apoptotic protein Bcl‐2 in IEC‐6 cells using Western blotting (Figure 3D). After irradiation, Bax levels in IEC‐6 cells remarkably increased (*P* < 0.01), whereas Bcl‐2 levels significantly decreased (*P* < 0.05). However, **10a** treatment dramatically alleviated the increased abundance of Bax (*P* < 0.05) and enhanced that of Bcl‐2 (*P* < 0.05) compared with the IR group (Figure 3D). These results indicated that **10a** can suppress IR‐induced apoptosis in IEC‐6 cells by regulating the apoptotic pathway.

### 3.7 Compound 10a improved the survival of TBI‐ and ABI‐exposed mice

**FIGURE 4:**
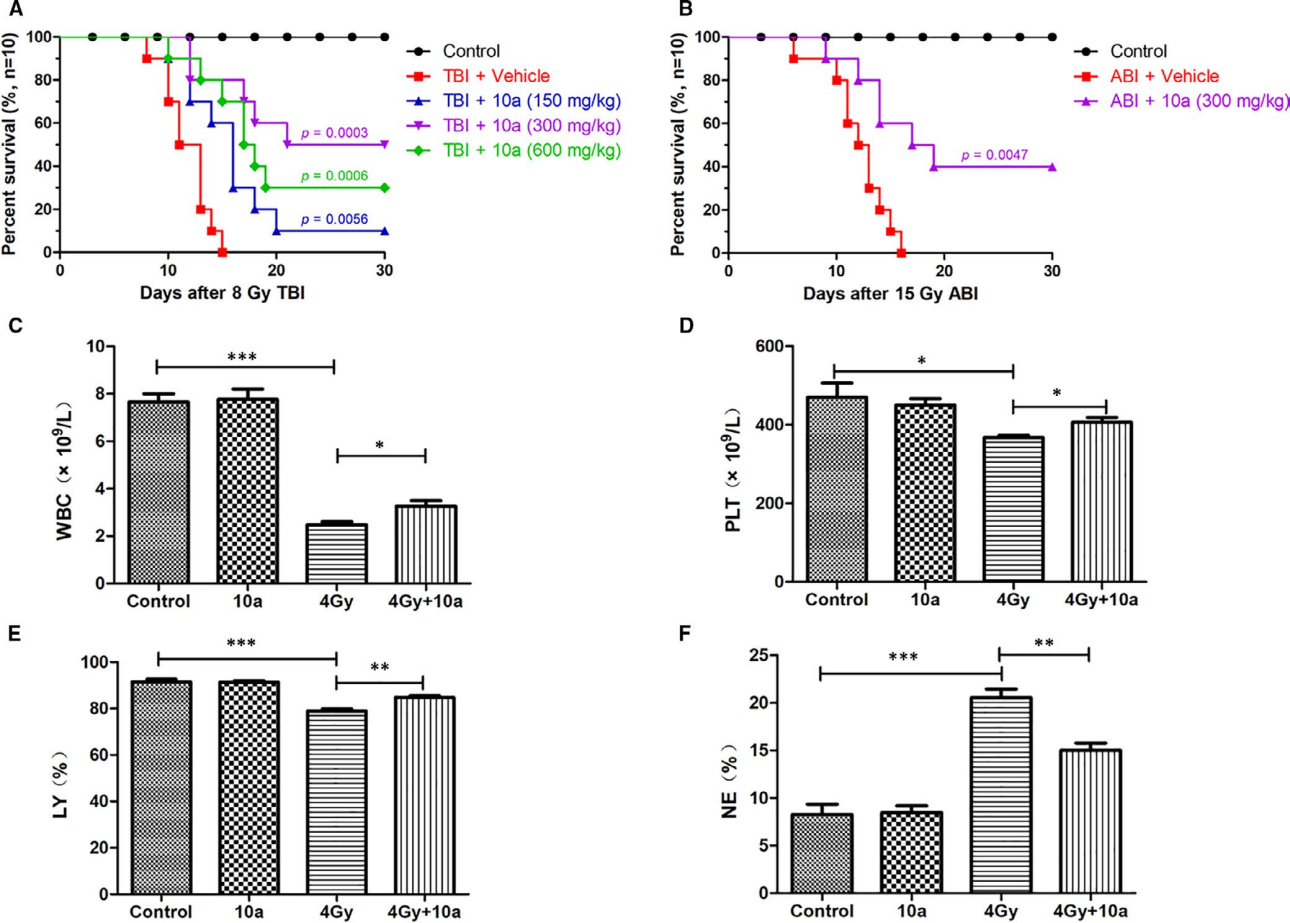
Compound **10a** increased the survival rates of mice and ameliorated TBI‐induced myelosuppression and myeloid skewing after irradiation. A, Survival analysis of mice exposed to 8.0 Gy TBI. All doses of **10a** treatment reduced mortality of mice following TBI compared with vehicle treatment (*P* < 0.05, n = 10). B, Survival analysis of mice exposed to 15.0 Gy ABI (*P* = 0.0047 **10a** vs vehicle, n = 10). C, WBC counts, D, PLT counts, E, NE% and F, LY% in peripheral blood were analysed 14 days after 4.0 Gy TBI, data are presented as the mean ± SEM (n = 5). *P < 0.05, **P < 0.01, ***P < 0.001

As illustrated in Figure 4A, all mice in the TBI +vehicle group died within 15 days, but no death was observed in the control group. All doses of **10a** treatment increased the survival rate of mice compared with the vehicle treatment. The survival rate in the group pretreated with 300 mg/kg **10a** was 50% (*P* = 0.0003 vs. TBI + vehicle group), whereas that in the 600 mg/kg **10a** group was only 30% (*P* = 0.0006 vs. TBI + vehicle group), indicating that a higher dose of **10a** may have toxic effects on mice. Consequently, a dose of 300 mg/kg was used for subsequent in vivo experiments.

In Figure 4B, all mice in the ABI +vehicle group died within 16 days. Particularly, the survival rate of mice administered with 300 mg/kg **10a** before radiation exposure was 40% (*P* = 0.0047 vs. ABI + vehicle group). These findings suggested that **10a** could effectively protect against irradiation‐induced intestinal damage and improve survival after a fatal dose of ABI.

### 3.8 Compound 10a ameliorated myelosuppression and myeloid skewing of TBI‐exposed mice

TBI can cause myelosuppression, and thus result in significant reduction in peripheral blood cell counts.^30^ IR may also affect the differentiation of HSCs, leading to myeloid skewing in peripheral blood.^36^, ^37^ As illustrated in Figure 4C‐F, compared with the control group, the mice irradiated with 4.0 Gy TBI exhibited a significant reduction in WBCs, PLTs and LY% in peripheral blood (*P* < 0.05), but a substantial increase in NE% (*P* < 0.001). Conversely, **10a** treatment notably reversed the changes in these cells in irradiated mice, demonstrating that **10a** could effectively improve the recovery of irradiated mice from radiation‐induced myelosuppression and myeloid skewing.

### 3.9 Compound 10a attenuated TBI‐induced bone marrow hematopoietic cell reduction

**FIGURE 5:**
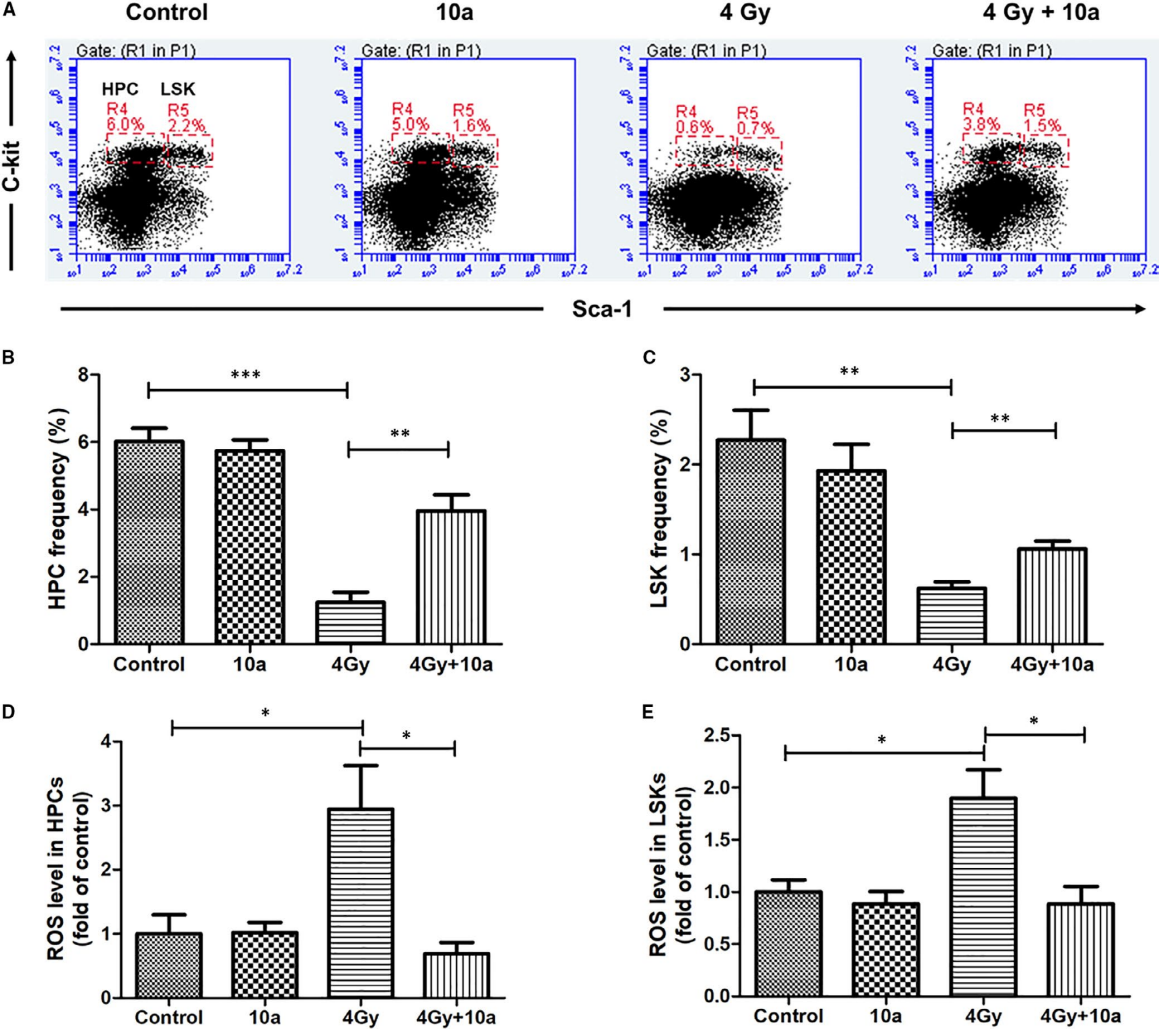
Compound **10a** attenuated TBI‐induced hematopoietic system injury. A, Representative fluorescence‐activated cell sorting plots of HPCs and LSKs. B, HPC and C, LSK frequencies in Lineage^‐^ bone marrow cells, and the intracellular ROS levels of D, HPCs and E, LSKs were analysed 14 d after TBI. Data are presented as the mean ± SEM (n = 5). *P < 0.05, **P < 0.01, ***P < 0.001

TBI may damage the hematopoietic tissue and deplete hematopoietic stem and progenitor cells (HSPCs). To investigate whether **10a** treatment could promote the recovery of the hematopoietic cells, we studied the effects of **10a** on LSK and HPC frequencies in the bone marrow cells of mice 14 days after exposure to 4.0 Gy TBI (Figure 5A). As demonstrated in Figure 5B and C, compared with the control group, the TBI + vehicle group showed a significant reduction in the frequencies of LSKs and HPCs (*P* < 0.01), whereas **10a** treatment dramatically increased the LSK and HPC frequencies (*P* < 0.01). These findings suggested that **10a** could prevent against radiation‐induced HSPC injury.

### 3.10 Compound 10a decreased TBI‐induced oxidative stress in the hematopoietic system

As we already demonstrated that **10a** could scavenge ROS at the cellular level, we proceeded to assess its effect on IR‐induced ROS levels in vivo. In Figure 5D and E, the ROS levels in LSKs and HPCs of irradiated mice increased dramatically compared with those of the control (*P* < 0.05), whereas **10a** treatment markedly reduced these ROS levels (*P* < 0.05). These results suggested that **10a** could scavenge ROS and consequently attenuate oxidative stress in irradiated HSPCs.

### 3.11 Compound 10a reduced intestinal damage of ABI‐exposed mice

**FIGURE 6:**
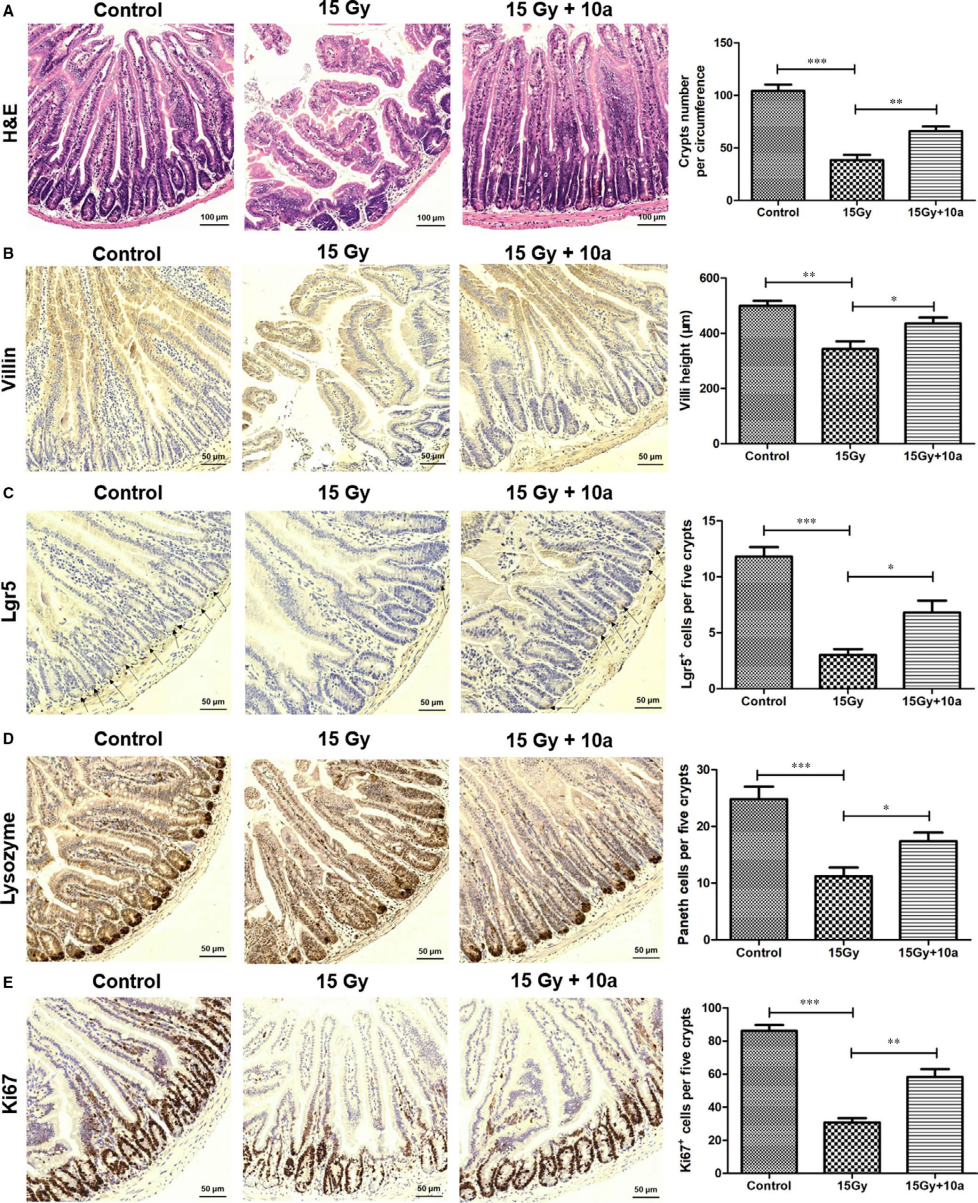
Compound **10a** promoted the structural repair of mouse small intestine and enhanced the regenerative ability of Lgr5^+^ ISCs after ABI. A, Representative HE‐stained sections of small intestinal structure. Scale bar: 100 μm. Quantification of crypts number in intestinal sections are shown. B, Representative immunohistochemistry images show the expression of villi in intestinal sections. Scale bar: 50 μm. Quantification of villi height in intestinal sections are shown. Representative immunohistochemistry images show the expression of C, Lgr5, D, lysozyme, and E, Ki67 in intestinal sections. Scale bar: 50 μm. Statistical analysis of C, Lgr5^+^ cells, D, Paneth cells, and E, Ki67^+^ cells are shown. Data are presented as the mean ± SEM (n = 5). *P < 0.05, **P < 0.01, ***P < 0.001

IR can induce necrosis and apoptosis in small intestinal epithelial cells, block the renewal of the villus epithelium, and lead to the loss of intestinal mucosal barrier function.^12^, ^38^ The morphological changes in the mouse small intestine are shown in Figure 6A. Five days after exposure to 15.0 Gy ABI, the mice in the vehicle group had significantly decreased villi height and number of crypts than the control (*P* < 0.01). Compared with the irradiated mice, those treated with **10a** exhibited increased number of crypts and villi height (Figure 6A, B). The expression of villin^+^ enterocytes was also decreased by ABI but increased by **10a** pretreatment (Figure 6B). These findings suggested that **10a** could prevent intestinal villus‐crypt structural damage in irradiated mice.

### 3.12 Compound 10a enhanced the regenerative ability of Lgr5^+^ intestinal stem cells (ISCs) and maintained homeostasis in intestinal cells of ABI‐exposed mice

The intestinal epithelium constantly self‐renews and can quickly regenerate after injury.^39^, ^40^, ^41^ Lgr5 is a marker for intestinal stem cells, and Lgr5^+^ ISCs have been shown to be essential for intestinal regeneration after radiation injury.^12^, ^42^, ^43^ Paneth cells are intestinal epithelium cells that secrete abundant antibacterial proteins including lysozyme, which are important for maintaining intestinal homeostasis.^39^ Ki67 is a marker for the regenerating epithelium, and can be used to determine the fraction of proliferating crypts.^12^ Therefore, we evaluated the effects of **10a** on ABI‐induced intestinal damage by analysing the numbers of Lgr5^+^ ISCs, lysozyme^+^ Paneth cells, and Ki67^+^ transient amplifying cells (TACs) using immunohistochemistry (Figure 6C‐E). Five days after ABI, the numbers of Lgr5^+^ ISCs, lysozyme^+^ cells, and Ki67^+^ TACs were notably reduced in the ABI + vehicle group compared with the control (*P* < 0.001). However, these changes were markedly reversed by **10a** treatment (*P* < 0.05), indicating that **10a** could enhance the regenerative ability of ISCs and maintain intestinal homeostasis by stimulating the proliferation and differentiation of intestinal crypt cells.

### 3.13 Compound 10a attenuated DNA damage in the small intestine of ABI‐exposed mice

**FIGURE 7:**
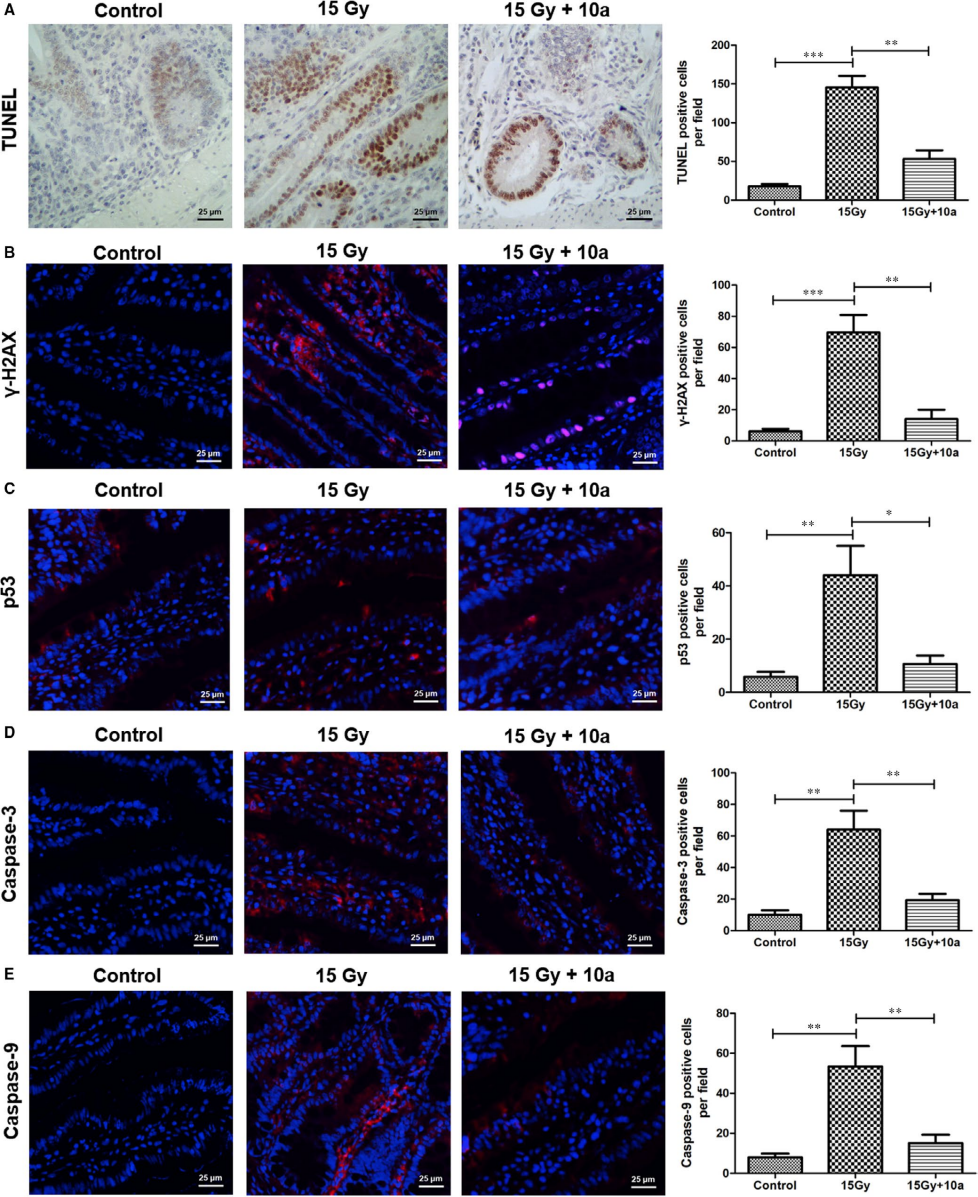
Compound **10a** attenuated ABI‐induced apoptosis and DNA damage in irradiated mice. A, Apoptosis was analysed using TUNEL assay. Quantification of TUNEL‐positive cells in the intestinal sections are shown. Representative immunofluorescence images show the levels of B, γ‐H2AX, C, p53, D, caspase‐3, and E, caspase‐9 in the intestinal sections. Red areas correspond to the proteins, whereas blue areas represent DAPI. Statistical analysis of B, γ‐H2AX‐positive cells, C, p53‐positive cells, D, caspase‐3‐positive cells, and E, caspase‐9‐positive cells are shown. Data are presented as the mean ± SEM (n = 5). *P < 0.05, **P < 0.01, ***P < 0.001. Scale bar: 25 μm

Phosphorylation of H2AX at Serine 139 (γ‐H2AX) is a marker for DNA double‐strand breaks (DSBs).^12^, ^44^ To investigate whether **10a** could mitigate ABI‐induced DSBs, we analysed the levels of γ‐H2AX in the small intestine using an immunofluorescence method. As illustrated in Figure 7B, immunofluorescence revealed a significant increase in the expression of γ‐H2AX in the irradiated mice compared with the control (*P* < 0.001), and, **10a** treatment significantly inhibited the phosphorylation of H2AX in the mouse small intestine (*P* < 0.01). These results suggested that **10a** can reduce DSBs in the small intestines of irradiated mice.

### 3.14 Compound 10a reduced the apoptotic rate in the small intestine of ABI‐exposed mice

We used the TUNEL assay to evaluate the effect of **10a** on the apoptosis of small intestinal tissues. Figure 7A show an increase number of apoptotic cells in the irradiated mice than in the control (*P* < 0.001), whereas **10a** treatment dramatically decreased the number of apoptotic cells (*P* < 0.01). These results suggested that **10a** could play a positive role in the inhibition of apoptosis. We further studied the effects of **10a** on ABI‐induced apoptosis using immunofluorescence assay. In the ABI + vehicle group, the levels of p53 (Figure 7C), caspase‐3 (Figure 7D) and caspase‐9 (Figure 7E) in the intestinal cells were significantly higher than those in the control (*P* < 0.01). In the **10a** treatment group, the levels of these proteins decreased significantly compared with those in the ABI + vehicle group (*P* < 0.05). Taken together, these data demonstrated that **10a** treatment could protect mice from ABI‐induced intestinal injuries by suppressing radiation‐induced apoptosis.

## 4 DISCUSSION

In this study, we designed and synthesized a series of hybrid compounds containing edaravone analogue and NBP ring‐opened derivatives and evaluated their radioprotective effects in vitro and in vivo. The anionic form of edaravone can eliminate free radicals through a single‐electron transfer mechanism.^28^ Owing to the electron‐withdrawing ability of the fluorine substituent on the benzene ring, the edaravone analogue moiety of these compounds can exhibit higher free‐radical scavenging activities.^28^ In addition, analysis of structure–activity relationships (SAR) showed that different substituted amines in the side chain of the ring‐opening NBP could have different effects on the solubility and permeability of the parent compound, which might affect the release of the edaravone analogue and NBP, and thus lead to varying bioactivities of these compounds. We observed that compound **10a**, which bears a dimethylamino moiety in side chain of ring‐opened NBP, displayed the highest radioprotective activities in IEC‐6 and HFL‐1 cells among all tested compounds. We speculate that the enhanced solubility of compound **10a** (LogP = 4.97) contributed to its good cell permeability. Besides, its higher biological activity than those of **10e** and **10f** may be due to the relatively low molecular weight and high absorbability of **10a**.

Since the hematopoietic system is the most radiosensitive system in the body, we investigated whether **10a** could prevent TBI‐induced hematopoietic tissue damage. We found that the oral administration of **10a** not only enhanced the survival of mice exposed to a fatal dose of TBI, but also mitigated TBI‐induced hematopoietic tissue damage. Furthermore, **10a** provided protection against radiation‐induced myelosuppression and myeloid skewing in mice by increasing WBCs, PLTs and LY%, and decreasing NE%. We also observed that **10a** consumption significantly increased the proportions of HSCs and HPCs in TBI mice. Overall, these results suggested that **10a** could ameliorate hematopoietic injury by promoting the recovery of HSC density and function.

Radiotherapy for abdominal and pelvic malignant tumours may injure the GI system, and the small intestine is particularly sensitive to the damaging effects of IR.^45^, ^46^ To evaluate whether **10a** could function as a radioprotector to treat radiation‐induced intestinal injury, we established an ABI mouse model and found that **10a** improved the survival of mice and preserved the intestinal crypt‐villus structure following 15.0 Gy ABI. The intestinal epithelium is one of the most rapidly self‐renewing tissues in mammals.^40^ ISCs, specifically marked by Lgr5, are indispensable for maintaining epithelial homeostasis and promoting structural regeneration. Lgr5^+^ ISCs can differentiate into Paneth cells, which can produce lysozymes. Our findings revealed that **10a** treatment increased the number of Lgr5^+^ cells and lysozymes^+^ Paneth cells after irradiation. Additionally, the abundance of Ki67, a proliferative marker of epithelial layer, was promoted in the mice pretreated with **10a**. Therefore, we concluded that **10a** might exert protective effects on ABI‐induced intestinal damage and could maintain the regenerative capacity of ISCs by stimulating the proliferation and differentiation of intestinal epithelial cells.

**FIGURE 8:**
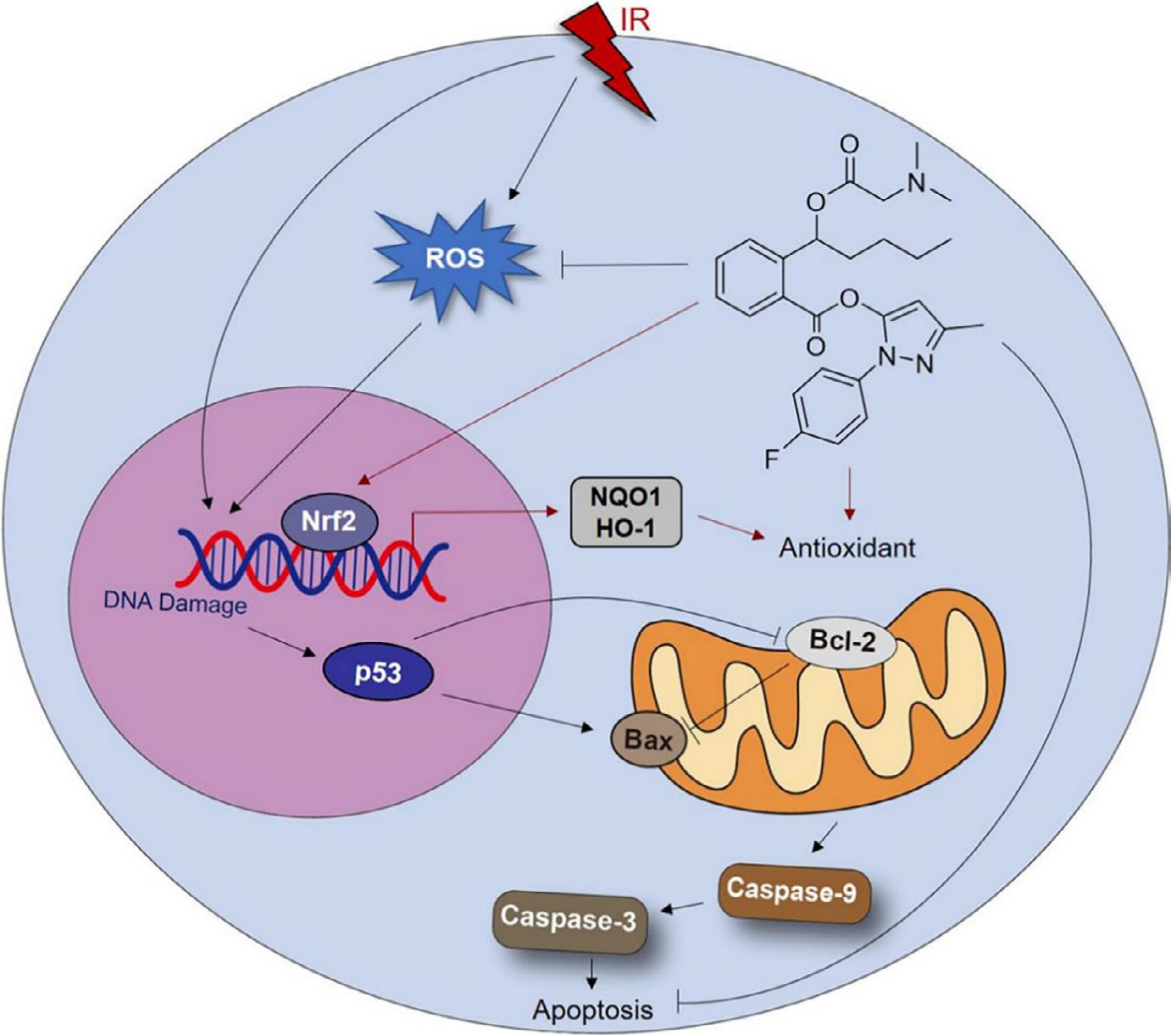
Proposed mechanism underlying the radioprotective effect of **10a**

Oxidative stress due to ROS accumulation after radiation exposure can contribute to tissue damage. We found that the ROS levels in irradiated cells were decreased by **10a**, indicating that **10a** can scavenge ROS generated by ionizing radiation. Besides, results showed that **10a** increased the expression levels of Nrf2, NQO1 and HO‐1 in IEC‐6 cells after irradiation, suggesting that **10a** may target Nrf2 and promote the production of antioxidant enzymes to reduce oxidative stress. Nevertheless, the levels of these proteins were not obviously increased by **10a** in unirradiated cells. It is probably because the cells are more sensitive to the stimulation of ionizing radiation compared with the effects of **10a** alone. Overall, **10a** can reduce oxidative stress in irradiated cells by directly scavenging ROS and activating the Nrf2 pathway (Figure 8).

DNA damage can be caused by radiant energy deposition and oxidative stress. Among the DNA injuries induced by irradiation, DSBs are the most fatal to cells and could lead to chromosome breakage and cell apoptosis. To quantify DSBs, γ‐H2AX is widely used as a biomarker.^12^, ^29^ We observed that γ‐H2AX levels in the small intestines of irradiated mice were reduced by **10a**, suggesting that **10a** could alleviate DNA damage by reducing oxidative stress in irradiated cells. Furthermore, the pro‐apoptotic molecule p53 is mainly activated by DNA strand breaks and can activate genes that regulate radiation‐induced DNA damage and repair, and cell apoptosis.^47^ Studies have shown that p53 promotes apoptosis by regulating the expression of Bcl‐2 protein family members, such as Bax, Bak, and Bcl‐2, in the cytoplasm.^2^, ^42^ We observed decreased p53 levels and Bax levels, and increased Bcl‐2 levels in irradiated cells after **10a** treatment. Moreover, previous studies have indicated that apoptosis is induced by mitochondrial outer membrane permeabilisation, which is affected by the Bcl‐2 family members.^48^, ^49^ After the release of cytochrome C and the formation of apoptosome, apoptosis is executed by a series of sequentially activated caspases, which can be categorized into initiator caspases (caspase‐8 and −9) and effector caspases (caspase‐3, −6 and −7).^42^, ^50^ We found that **10a** treatment downregulated the expression of caspase‐3 and caspase‐9 after irradiation. Together, these results suggested that **10a** could mitigate DNA damage induced by oxidative stress, thereby reduce apoptosis through the p53 pathway (Figure 8).

In summary, hybrid compounds with edaravone analogue and NBP ring‐opened derivatives were synthesized in the present study. Among these, compound **10a** displayed the most potent radioprotective activity in vitro. We showed that **10a** effectively increased the survival rates of irradiated mice, prevented TBI‐induced HSC injury, improved the structure and function of the small intestine, and decreased apoptosis of intestinal epithelium cells in ABI‐exposed mice. Our results proved that **10a** could reduce IR‐induced oxidative stress and DNA damage both in vitro and in vivo. Moreover, **10a** might mitigate IR‐induced intestinal damage via the p53‐dependent apoptotic pathway. We demonstrate that compound **10a** is a promising radioprotector, and the detailed molecular mechanisms underlying its radioprotective effects should be further studied.

## CONFLICT OF INTEREST

The authors declare that they have no conflict of interest.

## AUTHOR CONTRIBUTIONS

**Xuejiao Li:** Investigation (lead); Writing–original draft (lead). **Xinxin Wang:** Investigation (supporting). **Longfei Miao:** Investigation (supporting). **Yahong Liu:** Writing–review and editing (supporting). **Xiaona Lin:** Writing‐review and editing (supporting). **Yuying Guo:** Software (supporting). **Renbin Yuan:** Software (supporting). **Hongqqi Tian:** Project administration (lead); Writing‐review and editing (supporting).

## Supporting information

Supplementary Material

## Data Availability

The data used to support the findings of this study are available from the corresponding author upon request.

## References

[1] Patyar RR , Patyar S . Role of drugs in the prevention and amelioration of radiation induced toxic effects. Eur J Pharmacol. 2018;819:207‐216. 29221951 10.1016/j.ejphar.2017.12.011

[2] Feng T , Liu J , Zhou N , et al. CLZ‐8, a potent small‐molecular compound, protects radiation‐induced damages both in vitro and in vivo. Environ Toxicol Pharmacol. 2018;61:44‐51. 29852368 10.1016/j.etap.2018.05.004

[3] Zhou N , Feng T , Shen X , et al. Synthesis, characterization, and radioprotective activity of α, β‐unsaturated aryl sulfone analogs and their Tempol conjugates. Medchemcomm. 2017;8(5):1063‐1068. 30108819 10.1039/c7md00017k PMC6072343

[4] Tang L , Peng T , Wang G , et al. Synthesis and radioprotective effects of novel benzyl naphthyl sulfoxide (sulfone) derivatives transformed from Ex‐RAD. Medchemcomm. 2018;9(4):625‐631. 30108953 10.1039/c7md00573c PMC6072350

[5] Oh YJ , Kwak MS , Sung MH . Protection of radiation‐induced DNA damage by functional cosmeceutical poly‐gamma‐glutamate. J Microbiol Biotechnol. 2018;28(4):527‐533. 29385660 10.4014/jmb.1712.12016

[6] Zhang YR , Wang JY , Li YY , et al. Design and synthesis a mitochondria‐targeted dihydronicotinamide as radioprotector. Free Radic Biol Med. 2019;136:45‐51. 30946960 10.1016/j.freeradbiomed.2019.03.038

[7] Yang HJ , Youn H , Seong KM , et al. Psoralidin, a dual inhibitor of COX‐2 and 5‐LOX, regulates ionizing radiation (IR)‐induced pulmonary inflammation. Biochem Pharmacol. 2011;82(5):524‐534. 21669192 10.1016/j.bcp.2011.05.027

[8] Li H , Yang ZY , Liu C , et al. PEGylated ceria nanoparticles used for radioprotection on human liver cells under γ‐ray irradiation. Free Radic Biol Med. 2015;87:26‐35. 26117316 10.1016/j.freeradbiomed.2015.06.010

[9] Venkateswaran K , Shrivastava A , Agrawala PK , et al. Mitigation of radiation‐induced hematopoietic injury by the polyphenolic acetate 7, 8‐diacetoxy‐4‐methylthiocoumarin in mice. Sci Rep. 2016;6:37305. 27849061 10.1038/srep37305 PMC5110976

[10] Xue XL , Han XD , Li Y , et al. Astaxanthin attenuates total body irradiation‐induced hematopoietic system injury in mice via inhibition of oxidative stress and apoptosis. Stem Cell Res Ther. 2017;8(1):7. 28115023 10.1186/s13287-016-0464-3 PMC5260077

[11] Leibowitz BJ , Qiu W , Liu H , et al. Uncoupling p53 functions in radiation‐induced intestinal damage via PUMA and p21. Mol Cancer Res. 2011;9(5):616‐625. 21450905 10.1158/1541-7786.MCR-11-0052 PMC3096742

[12] Hou Q , Liu L , Dong Y , et al. Effects of thymoquinone on radiation enteritis in mice. Sci Rep. 2018;8(1):15122. 30310156 10.1038/s41598-018-33214-3 PMC6181979

[13] Tiwari V , Kamran MZ , Ranjan A , et al. Akt1/NFκB signaling pathway activation by a small molecule DMA confers radioprotection to intestinal epithelium in xenograft model. Free Radic Biol Med. 2017;108:564‐574. 28435051 10.1016/j.freeradbiomed.2017.04.029

[14] Singh VK , Seed TM . A review of radiation countermeasures focusing on injury‐specific medicinals and regulatory approval status: part I. Radiation sub‐syndromes, animal models and FDA‐approved countermeasures. Int J Radiat Biol. 2017;93(9):851‐869. 28650707 10.1080/09553002.2017.1332438

[15] Elwan AM , Salama AA , Sayed AM , et al. Biophysical and biochemical roles of *Moringa oleifera* leaves as radioprotector. Prog Biophys Mol Biol. 2018;140:142‐149. 29885346 10.1016/j.pbiomolbio.2018.06.003

[16] Hata K , Urushibara A , Yamashita S , et al. Chemical repair activity of free radical scavenger edaravone: reduction reactions with dGMP hydroxyl radical adducts and suppression of base lesions and AP sites on irradiated plasmid DNA. J Radiat Res. 2015;56(1):59‐66. 25212600 10.1093/jrr/rru079 PMC4572592

[17] Chen L , Liu Y , Dong L , et al. Edaravone protects human peripheral blood lymphocytes from γ‐irradiation‐induced apoptosis and DNA damage. Cell Stress Chaperones. 2015;20(2):289‐295. 25181965 10.1007/s12192-014-0542-3 PMC4326394

[18] Basol N , Aygun H , Gul SS . Beneficial effects of edaravone in experimental model of amitriptyline‐induced cardiotoxicity in rats. Naunyn Schmiedebergs Arch Pharmacol. 2019;392(11):1447‐1453. 31273393 10.1007/s00210-019-01683-6

[19] Zhang M , Teng CH , Wu FF , et al. Edaravone attenuates traumatic brain injury through anti‐inflammatory and anti‐oxidative modulation. Exp Ther Med. 2019;18(1):467‐474. 31281440 10.3892/etm.2019.7632 PMC6580098

[20] Parikh A , Kathawala K , Tan CC , et al. Self‐nanomicellizing solid dispersion of edaravone: part I ‐ oral bioavailability improvement. Drug Des Devel Ther. 2018;12:2051‐2069. 10.2147/DDDT.S161940 PMC6038876 30013324

[21] Wang X , Li Y , Zhao Q , et al. Design, synthesis and evaluation of nitric oxide releasing derivatives of 3‐*n*‐butylphthalide as antiplatelet and antithrombotic agents. Org Biomol Chem. 2011;9(16):5670‐5681. 21706121 10.1039/c1ob05478c

[22] Zhang X , Li Y , Li X , et al. Neuroprotective effect of Dl‐3‐*n*‐butylphthalide on patients with radiation‐induced brain injury: a clinical retrospective cohort study. Int J Neurosci. 2017;127(12):1059‐1064. 28332424 10.1080/00207454.2017.1310727

[23] Wang X , Wang L , Li T , et al. Novel hybrids of optically active ring‐opened 3‐*n*‐butylphthalide derivative and isosorbide as potential anti‐ischemic stroke agents. J Med Chem. 2013;56(7):3078‐3089. 23509954 10.1021/jm4001693

[24] Lan Z , Xu X , Xu W , et al. Discovery of 3‐*n*‐butyl‐2,3‐dihydro‐*1H*‐isoindol‐1‐one as a potential anti‐ischemic stroke agent. Drug Des Devel Ther. 2015;9:3377‐3391. 10.2147/DDDT.S84731 PMC4492651 26170623

[25] Yin W , Lan L , Huang Z , et al. Discovery of a ring‐opened derivative of 3‐*n*‐butylphthalide bearing NO/H_2_S‐donating moieties as a potential anti‐ischemic stroke agent. Eur J Med Chem. 2016;115:369‐380. 27031213 10.1016/j.ejmech.2016.03.044

[26] Abdoulaye IA , Guo YJ . A review of recent advances in neuroprotective potential of 3‐N‐Butylphthalide and its derivatives. Biomed Res Int. 2016;2016:5012341. 28053983 10.1155/2016/5012341 PMC5178327

[27] Hua K , Sheng X , Li TT , et al. The edaravone and 3‐*n*‐butylphthalide ring‐opening derivative 10b effectively attenuates cerebral ischemia injury in rats. Acta Pharmacol Sin. 2015;36(8):917‐927. 26073328 10.1038/aps.2015.31 PMC4564877

[28] Sheng X , Hua K , Yang C , et al. Novel hybrids of 3‐*n*‐butylphthalide and edaravone: design, synthesis and evaluations as potential anti‐ischemic stroke agents. Bioorg Med Chem Lett. 2015;25(17):3535‐3540. 26189079 10.1016/j.bmcl.2015.06.090

[29] Shao L , Luo Y , Zhou D . Hematopoietic stem cell injury induced by ionizing radiation. Antioxid Redox Signal. 2014;20(9):1447‐1462. 24124731 10.1089/ars.2013.5635 PMC3936513

[30] Han X , Zhang J , Xue X , et al. Theaflavin ameliorates ionizing radiation‐induced hematopoietic injury via the NRF2 pathway. Free Radic Biol Med. 2017;113:59‐70. 28939421 10.1016/j.freeradbiomed.2017.09.014

[31] Duru N , Gernapudi R , Zhang Y , et al. NRF2/miR‐140 signaling confers radioprotection to human lung fibroblasts. Cancer Lett. 2015;369(1):184‐191. 26300493 10.1016/j.canlet.2015.08.011 PMC4892935

[32] Riehl TE , Alvarado D , Ee X , et al. *Lactobacillus rhamnosus* GG protects the intestinal epithelium from radiation injury through release of lipoteichoic acid, macrophage activation and the migration of mesenchymal stem cells. Gut. 2019;68(6):1003‐1013. 29934438 10.1136/gutjnl-2018-316226 PMC7202371

[33] Gugliandolo A , Bramanti P , Mazzon E . Activation of Nrf2 by natural bioactive compounds: a promising approach for stroke? Int J Mol Sci. 2020;21(14):4875. 10.3390/ijms21144875 PMC7402299 32664226

[34] Kansanen E , Kuosmanen SM , Leinonen H , et al. The Keap1‐Nrf2 pathway: mechanisms of activation and dysregulation in cancer. Redox Biol. 2013;1:45‐49. 24024136 10.1016/j.redox.2012.10.001 PMC3757665

[35] Chang M , Xue J , Sharma V , et al. Protective role of hemeoxygenase‐1 in gastrointestinal diseases. Cell Mol Life Sci. 2015;72(6):1161‐1173. 25428780 10.1007/s00018-014-1790-1 PMC4342274

[36] Zhang J , Xue X , Han X , et al. Hydrogen‐rich water ameliorates total body irradiation‐induced hematopoietic stem cell injury by reducing hydroxyl radical. Oxid Med Cell Longev. 2017;2017:8241678. 28243358 10.1155/2017/8241678 PMC5294227

[37] Li K , Zhang J , Cao J , et al. 1,4‐Dithiothreitol treatment ameliorates hematopoietic and intestinal injury in irradiated mice: potential application of a treatment for acute radiation syndrome. Int Immunopharmacol. 2019;76:105913. 31627170 10.1016/j.intimp.2019.105913

[38] Zhang H , Yan H , Zhou X , et al. The protective effects of resveratrol against radiation‐induced intestinal injury. BMC Complement Altern Med. 2017;17(1):410. 28814292 10.1186/s12906-017-1915-9 PMC5559783

[39] Lu L , Jiang M , Zhu C , et al. Amelioration of whole abdominal irradiation‐induced intestinal injury in mice with 3,3'‐Diindolylmethane (DIM). Free Radic Biol Med. 2019;130:244‐255. 30352304 10.1016/j.freeradbiomed.2018.10.410

[40] Leibowitz BJ , Wei L , Zhang L , et al. Ionizing irradiation induces acute haematopoietic syndrome and gastrointestinal syndrome independently in mice. Nat Commun. 2014;5:3494. 24637717 10.1038/ncomms4494 PMC4327858

[41] Parker A , Maclaren OJ , Fletcher AG , et al. Cell proliferation within small intestinal crypts is the principal driving force for cell migration on villi. FASEB J. 2017;31(2):636‐649. 27811059 10.1096/fj.201601002 PMC5241155

[42] Cheng Y , Dong Y , Hou Q , et al. The protective effects of XH‐105 against radiation‐induced intestinal injury. J Cell Mol Med. 2019;23(3):2238‐2247. 30663222 10.1111/jcmm.14159 PMC6378229

[43] Liu W , Chen Q , Wu S , et al. Radioprotector WR‐2721 and mitigating peptidoglycan synergistically promote mouse survival through the amelioration of intestinal and bone marrow damage. J Radiat Res. 2015;56(2):278‐286. 25617317 10.1093/jrr/rru100 PMC4380048

[44] Pan MR , Peng G , Hung WC , et al. Monoubiquitination of H2AX protein regulates DNA damage response signaling. J Biol Chem. 2011;286(32):28599‐28607. 21676867 10.1074/jbc.M111.256297 PMC3151101

[45] Xiao HW , Li Y , Luo D , et al. Hydrogen‐water ameliorates radiation‐induced gastrointestinal toxicity via MyD88's effects on the gut microbiota. Exp Mol Med. 2018;50(1):e433. 29371696 10.1038/emm.2017.246 PMC5799803

[46] Fabbrizi MR , Meyer B , Misri S , et al. Transient PP2A inhibition alleviates normal tissue stem cell susceptibility to cell death during radiotherapy. Cell Death Dis. 2018;9(5):492. 29706648 10.1038/s41419-018-0559-0 PMC5924762

[47] Foster SS , De S , Johnson LK , et al. Cell cycle‐ and DNA repair pathway‐specific effects of apoptosis on tumor suppression. Proc Natl Acad Sci USA. 2012;109(25):9953‐9958. 22670056 10.1073/pnas.1120476109 PMC3382548

[48] Banjara S , Suraweera CD , Hinds MG , et al. The Bcl‐2 family: ancient origins, conserved structures, and divergent mechanisms. Biomolecules. 2020;10(1):128. 10.3390/biom10010128 PMC7022251 31940915

[49] Kalkavan H , Green DR . MOMP, cell suicide as a BCL‐2 family business. Cell Death Differ. 2018;25(1):46‐55. 29053143 10.1038/cdd.2017.179 PMC5729535

[50] Lei B , Zhou X , Lv D , et al. Apoptotic and nonapoptotic function of caspase 7 in spermatogenesis. Asian J Androl. 2017;19(1):47‐51. 26643564 10.4103/1008-682X.169563 PMC5227673

