# Supplementary material for: Synthesis and radioprotective effects of novel hybrid compounds containing edaravone analogue and 3‐n‐butylphthalide ring‐opening derivatives
Source: J Cell Mol Med. 2021 May 8;25(12):5470–85. doi: 10.1111/jcmm.16557 (PMC8184683; doi:10.1111/jcmm.16557)
Supplement: Supplementary file 1 — Supplementary Material [file JCMM-25-5470-s001.doc]

**Synthesis and radioprotective effects of** **novel hybrid compounds containing** **edaravone analogue and** **3-*n*-butylphthalide ring-opening derivatives**

Xuejiao Li^1^, Xinxin Wang^1^, Longfei Miao^1^, Yahong Liu^1^, Xiaona Lin^1^, Yuying Guo^1^, Renbin Yuan^1^, Hongqi Tian^1*^

^1^Tianjin Key Laboratory of Radiation Medicine and Molecular Nuclear Medicine, Institute of Radiation Medicine, Peking Union Medical College and Chinese Academy of Medical Science, Tianjin 300192, China

^*^Correspondence: tianhongqi@irm-cams.ac.cn

**Supporting Information**

**Experimental Section**

**Materials and methods**

**Cell viability assay**

IEC-6 and HFL-1 cells were plated in 96**-**well plates (5 × 10^3^ cells per well) and incubated overnight. The cells were treated with compound **10a** (10 μmol/L) at the given time, and irradiated with 8.0 or 10.0 Gy. After cultured for 24 h, cell viability was measured using the luminescence-based CellTiter Glo TM assay.

**Survival study**

The mice were randomly divided into 3 groups (*n* = 10 per group), namely control, TBI + vehicle, and TBI + **10a** (300 mg/kg). Mice in the irradiation groups received 8.0 Gy TBI. The mice of the TBI + **10a** group were treated with **10a** at the indicated dose 1 h after radiation. The survival status of each mouse was observed for 30 days after irradiation, and the results were expressed as survival rates.

**Results**

**Radioprotective activities of compound 10a in IEC-6 and HFL-1 cells**

We investigated the relationship between the administration time and radioprotective effect of compound **10a**. As shown in Figure S1, **10a** showed radioprotective effects in IEC-6 and HFL-1 cells when administrated 1 h before irradiation (*p* < 0.05). And the cell viabilities were not obviously increased by **10a** when administrated 1 h after irradiation.

**Compound 10a did not improve the survival of TBI-exposed mice**

As illustrated in Figure S2, all mice in the TBI + vehicle group died within 20 days, but no death was observed in the control group. Besides, all mice in the TBI + **10a** group died within 24 days, so **10a** did not significantly increase the survival rate of mice when administrated 1 h after irradiation (*p* > 0.05).

**Synthetic procedures Section**

**Synthesis of all intermediates, compound 9 and target compounds 10a–10f**

**2-(4-fluorophenyl)-5-methyl-2,4-dihydro-pyrazol-3-one (2).** Acetylacetic ether acetate (2.5 mL, 20.0 mmol) was added to a mixture solution of **1** (3.2 g, 20.0 mmol) and sodium acetate (1.7 g, 20.5 mmol) in acetic acid (20 mL). The reaction mixture was refluxed for 10 h and allowed to cool to room temperature. The mixture was added to a saturated aqueous solution of NaHCO_3_ until its pH value was adjusted to 7, and the solution was extracted with ethyl acetate. The combined organic layer was then dried over anhydrous Na_2_SO_4_, and evaporated to dryness. The resulting residue was purified using column chromatography (petroleum ether/EtOAc = 5:3, v/v) to afford compound **2** as a light-yellow solid (3.0 g, 78%). ^1^H NMR (400 MHz, CDCl_3_) *δ* = 8.06 – 7.71 (m, 2H), 7.26 – 6.93 (m, 2H), 3.45 (s, 2H), 2.21 (s, 3H); ESI-MS: *m/z* = 193 (M+H)^+^.

**3-butyl-3*H*-isobenzofuran-1-one (4, NBP).** To a solution of magnesium (4.8 g, 200.0 mmol) in anhydrous tetrahydrofuran (50 mL), 1-bromobutane (27.4 g, 200.0 mmol) was added under nitrogen atmosphere until the Grignard reaction was started. Then, the reaction mixture was refluxed for 1 h. After cooling, a solution of **3** (10.0 g, 66.7 mmol) in anhydrous tetrahydrofuran (200 mL) was added dropwise to the Grignard solution at -5 ℃. Next, the reaction mixture was stirred at room temperature for 5 h and the reaction was quenched by addition of a saturated solution of NH_4_Cl. The mixture was acidified with 1 M HCl until its pH value was adjusted to 7 and then stirred for 1 h at room temperature. The solution was extracted with ethyl acetate, dried, concentrated and the residue was purified using column chromatography (petroleum ether/EtOAc = 5:1, v/v) to give NBP as a light-yellow oil (7.9 g, 62%). ^1^H NMR (400 MHz, CDCl3) *δ* = 7.90 (d, *J* = 7.6 Hz, 1H), 7.78 – 7.62 (m, 1H), 7.60 – 7.39 (m, 2H), 5.48 (dd, *J* = 7.8, 4.1 Hz, 1H), 2.15 – 1.95 (m, 1H), 1.87 – 1.64 (m, 1H), 1.54 – 1.31 (m, 4H), 0.91 (t, *J* = 7.1 Hz, 3H); ESI-MS: *m/z* = 191 (M+H)^+^.

**2-(1-hydroxypentyl)benzoic acid (5, HPBA).** To a solution of NBP (7.9 g, 41.5 mmol) in CH_3_OH-H_2_O (80 mL, 1:1 v/v), NaOH (2.5 g, 62.3 mmol) was added and the reaction mixture was left stirring at reflux temperature for 1 h. The solvent was removed under reduced pressure and dissolved in water (40 mL), and the mixture was acidified with 1 M HCl to pH 3-4 at -5-0 ℃. The mixture was extracted with cold Et_2_O (20 mL × 3) to get HPBA and quickly used for the next step without any purification.

**2-(1-acetoxypentyl)benzoic acid (6).** A solution of acetyl chloride (0.6 mL, 7.8 mmol) in anhydrous dichloromethane (10 mL) was added dropwise to a mixture of HPBA (2.6 mmol), Et_3_N (1.1 mL, 7.8 mmol), and DMAP (37.0 mg, 0.3 mmol) in dichloromethane (30 mL) at -5 °C, and the solution was stirred at -5 °C for 5 h. The mixture was acidified with 1 M HCl to pH 2 and then stirred for 1 h at room temperature. The organic layer was separated, dried, and filtered. After removal of solvent, the residue was recrystallized from n-hexane to obtain compound **6** as a white crystal (0.4 g, 61%, over two steps). ^1^H NMR (400 MHz, CDCl_3_) *δ* = 8.06 (d, *J* = 7.9 Hz, 1H), 7.57 (d, *J* = 5.1 Hz, 2H), 7.46 – 7.33 (m, 1H), 6.63 (dd, *J* = 7.4, 4.0 Hz, 1H), 2.12 (s, 3H), 2.00 – 1.74 (m, 2H), 1.51 – 1.28 (m, 4H), 0.92 (t, *J* = 6.9 Hz, 3H); ESI-MS: *m/z* = 273 (M+Na)^+^.

**2-(1-(2-bromoacetoxy)pentyl)benzoic acid (7).** Compound **7** was synthesized in the same way as compound **6** except starting with HPBA (38.9 mmol) and 2-bromoacetyl bromide (10.2 mL, 116.7 mmol) and was obtained as a white crystal (6.5 g, 51%, over two steps). ^1^H NMR (400 MHz, CDCl_3_) *δ* = 8.08 (d, *J* = 7.8 Hz, 1H), 7.60 (d, *J* = 4.0 Hz, 2H), 7.41 (dd, *J* = 7.8, 4.0 Hz, 1H), 6.73 (s, 1H), 3.88 (s, 2H), 1.91 (d, *J* = 5.1 Hz, 2H), 1.60 – 1.30 (m, 4H), 0.93 (t, *J* = 7.0 Hz, 3H); ESI-MS: *m/z* = 327 (M-H)^-^.

***General procedure for the synthesis of compounds 8a–8f***

To a solution of compound **7** (1.0 g, 3.0 mmol) and K_2_CO_3_ (0.4 g, 3.0 mmol) in acetonitrile (20 mL), the corresponding amine was added, and the solution was left stirring at room temperature for 8-12 h. The solution was then filtered, and the solvent was removed under reduced pressure. The amino derivatives were purified using flash chromatography (CH_2_Cl_2_/MeOH = 40/1-20/1, v/v) to give compounds **8a–8f** as light-yellow oil (49-71%).

**2-(1-(2-dimethylaminoacetoxy)pentyl)benzoic acid (8a).** The title compound was obtained as a light-yellow oil (59%). ^1^H NMR (400 MHz, CDCl_3_) *δ* = 7.81 (d, *J* = 7.7 Hz, 1H), 7.41 (q, *J* = 7.6 Hz, 2H), 7.33 – 7.19 (m, 1H), 6.80 – 6.53 (m, 1H), 3.64 (d, *J* = 16.7 Hz, 1H), 3.52 – 3.35 (m, 1H), 2.56 (s, 6H), 1.89 (d, *J* = 4.2 Hz, 2H), 1.43 – 1.30 (m, 4H), 0.86 (d, *J* = 6.9 Hz, 3H); ESI-MS: *m/z* = 294 (M+H)^+^.

**2-(1-(2-diethylaminoacetoxy)pentyl)benzoic acid (8b).** The title compound was obtained as a light-yellow oil (62%). ^1^H NMR (400 MHz, CDCl_3_) *δ* = 7.78 (d, *J* = 7.6 Hz, 1H), 7.41 (t, *J* = 6.1 Hz, 2H), 7.33 – 7.22 (m, 1H), 6.61 (t, *J* = 6.0 Hz, 1H), 3.64 (dd, *J* = 61.6, 17.0 Hz, 2H), 3.04 – 2.80 (m, 4H), 1.91 (d, *J* = 6.5 Hz, 2H), 1.34 (dd, *J* = 16.1, 6.3 Hz, 4H), 1.16 (t, *J* = 7.2 Hz, 6H), 0.87 (t, *J* = 7.0 Hz, 3H); ESI-MS: *m/z* = 322 (M+H)^+^.

**2-(1-(2-(pyrrolidin-1-yl)acetoxy)pentyl)benzoic acid (8c).** The title compound was obtained as a light-yellow oil (71%). ^1^H NMR (400 MHz, CDCl_3_) *δ* = 7.76 (d, *J* = 7.5 Hz, 1H), 7.45 – 7.32 (m, 2H), 7.25 (dd, *J* = 14.6, 7.7 Hz, 1H), 6.71 – 6.54 (m, 1H), 3.95 – 3.48 (m, 2H), 3.09 – 2.87 (m, 4H), 1.91 (s, 6H), 1.41 – 1.19 (m, 4H), 0.84 (t, *J* = 7.0 Hz, 3H); ESI-MS: *m/z* = 320 (M+H)^+^.

**2-(1-(2-(piperidin-1-yl)acetoxy)pentyl)benzoic acid (8d).** The title compound was obtained as a light-yellow oil (67%). ^1^H NMR (400 MHz, CDCl_3_) *δ* = 7.76 (d, *J* = 7.6 Hz, 1H), 7.38 (d, *J* = 4.0 Hz, 2H), 7.25 (dd, *J* = 7.8, 4.0 Hz, 1H), 6.52 (s, 1H), 3.60 (d, *J* = 16.6 Hz, 1H), 3.25 (d, *J* = 16.6 Hz, 1H), 2.72 (d, *J* = 24.7 Hz, 4H), 1.99 – 1.79 (m, 2H), 1.78 – 1.59 (m, 4H), 1.48 (s, 3H), 1.37 – 1.20 (m, 4H), 0.84 (t, *J* = 7.0 Hz, 3H); ESI-MS: *m/z* = 334 (M+H)^+^.

**2-(1-(2-morpholin-4-yl-acetoxy)pentyl)benzoic acid (8e).** The title compound was obtained as a light-yellow oil (64%). ^1^H NMR (600 MHz, CDCl_3_) *δ* = 7.83 (d, *J* = 7.4 Hz, 1H), 7.55 – 7.35 (m, 2H), 7.32 – 7.29 (m, 1H), 6.81 (dd, *J* = 8.4, 4.3 Hz, 1H), 3.79 – 3.68 (m, 4H), 3.26 (dd, *J* = 40.0, 16.4 Hz, 2H), 2.65 – 2.52 (m, 4H), 2.01 – 1.78 (m, 2H), 1.48 – 1.28 (m, 4H), 0.89 (t, *J* = 7.0 Hz, 3H); ESI-MS: *m/z* = 336 (M+H)^+^.

**2-(1-(2-(4-methylpiperazin-1-yl)acetoxy)pentyl)benzoic acid (8f).** The title compound was obtained as a light-yellow oil (49%). ^1^H NMR (400 MHz, CDCl_3_) *δ* = 7.81 (d, *J* = 7.6 Hz, 1H), 7.40 (dt, *J* = 14.8, 7.2 Hz, 2H), 7.34 – 7.22 (m, 1H), 6.75 (dd, *J* = 7.8, 5.2 Hz, 1H), 3.32 (q, *J* = 16.8 Hz, 2H), 2.92 (d, *J* = 48.6 Hz, 8H), 2.61 (s, 3H), 1.89 (dd, *J* = 8.5, 5.1 Hz, 2H), 1.36 – 1.28 (m, 4H), 0.86 (t, *J* = 6.9 Hz, 4H); ESI-MS: *m/z* = 349 (M+H)^+^.

**1-(4-fluorophenyl)-3-methyl-1*H*-pyrazol-5-yl 2-(1-acetoxypentyl)benzoate (9).** To a solution of **6** (0.4 g, 1.6 mmol) in anhydrous dichloromethane (20 mL), oxalyl chloride (0.3 mL, 3.5 mmol) was added, and the solution was stirred at room temperature for 11 h. Then solvent of the mixture was evaporated to obtain the corresponding acyl chloride. A solution of compound **2** (0.3 g, 1.6 mmol) and Et_3_N (0.7 mL, 4.8 mmol) in anhydrous dichloromethane (20 mL) was stirred for 10 min at 0 ℃. Then the acyl chloride of **6** obtained above in anhydrous dichloromethane (20 mL) was added dropwise to the solution, the reaction mixture was stirred at room temperature for 4 h and then poured into water, extracted with ethyl acetate for three times. The combined organic phase was dried, filtered, and evaporated in vacuo. The residue was purified using column chromatography (petroleum ether/EtOAc = 9:1, v/v) to give compound **9** as a light-yellow solid (0.3 g, 44%). ^1^H NMR (400 MHz, CDCl_3_) *δ* = 7.92 (d, *J* = 7.6 Hz, 1H), 7.70 – 7.51 (m, 4H), 7.36 (ddd, *J* = 8.5, 6.5, 2.2 Hz, 1H), 7.14 (t, *J* = 8.6 Hz, 2H), 6.49 (t, *J* = 6.4 Hz, 1H), 6.27 (s, 1H), 2.38 (s, 3H), 2.09 (s, 3H), 1.79 (dd, *J* = 10.9, 4.3 Hz, 2H), 1.39 – 1.26 (m, 4H), 0.89 (t, *J* = 7.1 Hz, 3H); ESI-MS: *m/z* = 425 (M+H)^+^.

**1-(4-fluorophenyl)-3-methyl-1*H*-pyrazol-5-yl 2-(1-(2-dimethylaminoacetoxy)pentyl)benzoate (10a).** Compound **10a** was prepared using the same method as compound **9** except starting with compounds **8a** and **2** and was obtained as a light-yellow solid (63%). ^1^H NMR (400 MHz, CDCl_3_) *δ* = 7.93 (d, *J* = 7.6 Hz, 1H), 7.57 (dd, *J* = 10.0, 5.7 Hz, 4H), 7.45 – 7.33 (m, 1H), 7.14 (t, *J* = 8.6 Hz, 2H), 6.64 – 6.50 (m, 1H), 6.28 (s, 1H), 3.23 (q, *J* = 16.4 Hz, 2H), 2.38 (s, 3H), 2.36 (s, 6H), 1.85 – 1.78 (m, 2H), 1.42 – 1.30 (m, 4H), 0.89 (t, *J* = 7.1 Hz, 3H); ^13^C NMR (101 MHz, CDCl_3_) *δ* = 168.74, 161.75, 160.72, 159.30, 148.15, 144.39, 143.38, 132.81, 129.61, 126.52, 125.55, 124.54, 124.33, 124.24, 115.12, 114.89, 94.99, 71.78, 59.28, 44.09, 35.33, 26.99, 21.34, 13.49, 12.92; HRMS (ESI): *m/z* [M+H]^+^ calcd for C_26_H_31_FN_3_O_4_: 468.2293, found: 468.2293.

**1-(4-fluorophenyl)-3-methyl-1*H*-pyrazol-5-yl 2-(1-(2-diethylaminoacetoxy)pentyl) benzoate (10b).** Compound **10b** was prepared using the same method as compound **9** except starting with compounds **8b** and **2** and was obtained as a light-yellow solid (55%). ^1^H NMR (400 MHz, CDCl_3_) *δ* = 7.92 (d, *J* = 7.9 Hz, 1H), 7.58 (dt, *J* = 6.8, 5.3 Hz, 4H), 7.36 (ddd, *J* = 8.3, 5.9, 2.7 Hz, 1H), 7.13 (t, *J* = 8.6 Hz, 2H), 6.58 – 6.46 (m, 1H), 6.27 (s, 1H), 3.40 (d, *J* = 8.0 Hz, 2H), 2.67 (q, *J* = 7.2 Hz, 4H), 2.37 (s, 3H), 1.80 (t, *J* = 7.2 Hz, 2H), 1.34 (ddd, *J* = 31.5, 15.5, 9.1 Hz, 5H), 1.06 (t, *J* = 7.2 Hz, 6H), 0.88 (t, *J* = 7.0 Hz, 3H); ^13^C NMR (101 MHz, CDCl_3_) *δ* = 168.74, 160.12, 158.61, 151.73, 137.42, 132.78, 127.85, 121.98, 121.90, 114.80, 114.58, 108.43, 99.00, 46.75, 42.20, 37.90, 28.68, 26.95, 21.26, 12.92, 12.84, 11.84, 11.65, 10.91; HRMS (ESI): *m/z* [M+H]^+^ calcd for C_28_H_35_FN_3_O_4_: 496.2606, found: 496.2607.

**1-(4-fluorophenyl)-3-methyl-1*H*-pyrazol-5-yl 2-(1-(2-(pyrrolidin-1-yl)acetoxy)pentyl)benzoate (10c).** Compound **10c** was prepared using the same method as compound **9** except starting with compounds **8c** and **2** and was obtained as a light-yellow solid (61%). ^1^H NMR (400 MHz, CDCl_3_) *δ* = 7.92 (d, *J* = 7.7 Hz, 1H), 7.58 (ddd, *J* = 9.1, 5.4, 2.9 Hz, 4H), 7.35 (ddd, *J* = 8.5, 5.8, 2.8 Hz, 1H), 7.13 (t, *J* = 8.6 Hz, 2H), 6.55 (dd, *J* = 7.1, 5.8 Hz, 1H), 6.26 (s, 1H), 3.41 (d, *J* = 7.1 Hz, 2H), 2.66 (d, *J* = 3.5 Hz, 4H), 2.37 (s, 3H), 1.82 (dd, *J* = 7.8, 5.1 Hz, 6H), 1.44 – 1.29 (m, 4H), 0.88 (t, *J* = 7.1 Hz, 3H); ^13^C NMR (101 MHz, CDCl_3_) *δ* = 168.97, 162.63, 161.74, 160.20, 149.18, 145.21, 144.42, 133.89, 130.65, 127.60, 126.62, 125.35, 125.27, 116.13, 115.90, 96.01, 73.16, 55.94, 53.61, 36.33, 29.69, 27.96, 23.88, 22.35, 14.49, 13.91; HRMS (ESI): *m/z* [M+H]^+^ calcd for C_28_H_33_FN_3_O_4_: 494.2455, found: 494.2474.

**1-(4-fluorophenyl)-3-methyl-1*H*-pyrazol-5-yl 2-(1-(2-(piperidin-1-yl)acetoxy)pentyl)benzoate (10d).** Compound **10d** was prepared using the same method as compound **9** except starting with compounds **8d** and **2** and was obtained as a light-yellow solid (58%). ^1^H NMR (400 MHz, CDCl_3_) *δ* = 7.92 (d, *J* = 8.2 Hz, 1H), 7.70 – 7.52 (m, 4H), 7.36 (ddd, *J* = 8.5, 6.1, 2.6 Hz, 1H), 7.14 (t, *J* = 8.6 Hz, 2H), 6.61 – 6.43 (m, 1H), 6.27 (s, 1H), 3.27 (q, *J* = 16.4 Hz, 2H), 2.54 (d, *J* = 3.9 Hz, 4H), 2.38 (s, 3H), 1.85 – 1.75 (m, 2H), 1.67 – 1.60 (m, 4H), 1.47 – 1.42 (m, 2H), 1.40 – 1.30 (m, 4H), 0.89 (t, *J* = 7.1 Hz, 3H); ^13^C NMR (101 MHz, CDCl_3_) *δ* = 168.70, 161.81, 160.79, 159.36, 148.15, 144.50, 143.48, 132.76, 129.59, 126.49, 125.66, 124.63, 124.36, 124.28, 115.11, 114.89, 95.02, 71.76, 58.99, 53.02, 35.36, 26.98, 24.68, 22.78, 21.34, 13.47, 12.90; HRMS (ESI): *m/z* [M+H]^+^ calcd for C_29_H_35_FN_3_O_4_: 508.2612, found: 508.2603.

**1-(4-fluorophenyl)-3-methyl-1*H*-pyrazol-5-yl 2-(1-(2-morpholin-4-yl-acetoxy)pentyl)benzoate (10e).** Compound **10e** was prepared using the same method as compound **9** except starting with compounds **8e** and **2** and was obtained as a light-yellow solid (64%). ^1^H NMR (400 MHz, CDCl_3_) *δ* = 7.93 (d, *J* = 7.8 Hz, 1H), 7.69 – 7.49 (m, 4H), 7.47 – 7.32 (m, 1H), 7.14 (t, *J* = 8.6 Hz, 2H), 6.64 – 6.47 (m, 1H), 6.27 (s, 1H), 3.74 (t, *J* = 4.6 Hz, 4H), 3.26 (q, *J* = 16.5 Hz, 2H), 2.57 (d, *J* = 5.0 Hz, 4H), 2.38 (s, 3H), 1.82 (d, *J* = 6.5 Hz, 2H), 1.39 – 1.28 (m, 4H), 0.89 (t, *J* = 7.1 Hz, 3H); ^13^C NMR (101 MHz, CDCl_3_) *δ* = 168.28, 160.71, 148.17, 144.32, 132.83, 129.63, 126.58, 126.24, 125.53, 124.52, 124.35, 124.27, 119.67, 119.59, 115.13, 114.90, 114.60, 114.37, 95.00, 71.94, 65.66, 58.44, 52.16, 35.30, 26.96, 21.33, 13.47, 12.92; HRMS (ESI): *m/z* [M+H]^+^ calcd for C_28_H_33_FN_3_O_5_: 510.2404, found: 510.2399.

**1-(4-fluorophenyl)-3-methyl-1*H*-pyrazol-5-yl 2-(1-(2-(4-methylpiperazin-1-yl)acetoxy)pentyl)benzoate (10f).** Compound **10f** was prepared using the same method as compound **9** except starting with compounds **8f** and **2** and was obtained as a light-yellow solid (42%). ^1^H NMR (400 MHz, CDCl^3^) *δ* = 7.91 (d, *J* = 7.7 Hz, 1H), 7.64 – 7.48 (m, 4H), 7.43 – 7.31 (m, 1H), 7.12 (t, *J* = 8.6 Hz, 2H), 6.62 – 6.45 (m, 1H), 6.25 (s, 1H), 3.25 (q, *J* = 16.6 Hz, 2H), 2.63 (d, *J* = 11.2 Hz, 8H), 2.37 (d, *J* = 4.1 Hz, 6H), 1.80 (d, *J* = 6.4 Hz, 2H), 1.32 (ddd, *J* = 9.6, 7.7, 3.9 Hz, 4H), 0.86 (d, *J* = 7.1 Hz, 3H); ^13^C NMR (101 MHz, CDCl_3_) *δ* = 168.23, 160.73, 159.28, 148.16, 144.19, 143.34, 132.96, 129.65, 126.65, 125.52, 124.47, 124.31, 124.23, 115.12, 114.89, 94.99, 72.13, 57.47, 53.13, 49.83, 44.85, 35.28, 28.68, 26.94, 21.33, 13.50, 12.93; HRMS (ESI): *m/z* [M+H]^+^ calcd for C_29_H_36_FN_4_O_4_: 523.2721, found: 523.2715.

**Scheme S1** Synthesis of compounds **9** and **10a–10f**. Reagents and reaction conditions: (a) acetylacetic ether acetate, AcONa, AcOH, reflux, N_2_, 10 h; (b) (ⅰ) n-BuMgBr, THF, –5 ℃, 5 h; (ⅱ) 1 M HCl, RT, 1 h; (c) (ⅰ) NaOH, CH_3_OH-H_2_O, reflux, 1 h; (ⅱ) 1 M HCl, –5 to 0 ℃, 1 h; (d) CH_3_COCl, Et_3_N, DMAP, dry CH_2_Cl_2_, –5 ℃, 5 h; (e) BrCH_2_COBr, Et_3_N, DMAP, dry CH_2_Cl_2_, –5 ℃, 5 h; (f) corresponding amines, K_2_CO_3_, CH_3_CN, RT, 8–12 h; (g) (ⅰ) (COCl)_2_, dry CH_2_Cl_2_, RT, 11 h; (ⅱ) **2**, Et_3_N, dry CH_2_Cl_2_, RT, 4 h; (h) (ⅰ) (COCl)_2_, dry CH_2_Cl_2_, RT, 10–12 h; (ⅱ) **2**, Et_3_N, dry CH_2_Cl_2_, RT, 5–7 h.

**HPLC Method**

The compounds were chromatographed on a C18 column (250mm X 4.6mm, 5μm, Inertisl ODS-SP) with the column temperature of 25 ℃ and detected at 214 (254) nm. The mobile phases were A: water (15%), B: acetonitrile (85%) with flow rate of 1.0 mL/min.

**Table S1** The oil - water partition coefficient (LogP) values of test compounds*a*

| Compound | LogP |
| --- | --- |
| **9** | 5.43 |
| **10a** | 4.97 |
| **10b** | 5.75 |
| **10c** | 5.50 |
| **10d** | 5.89 |
| **10e** | 4.74 |
| **10f** | 4.66 |

*^a^*The LogP values of compounds **9** and **10a–10f** were calculated using the MOE 2015.10 software.

**Figures**

**
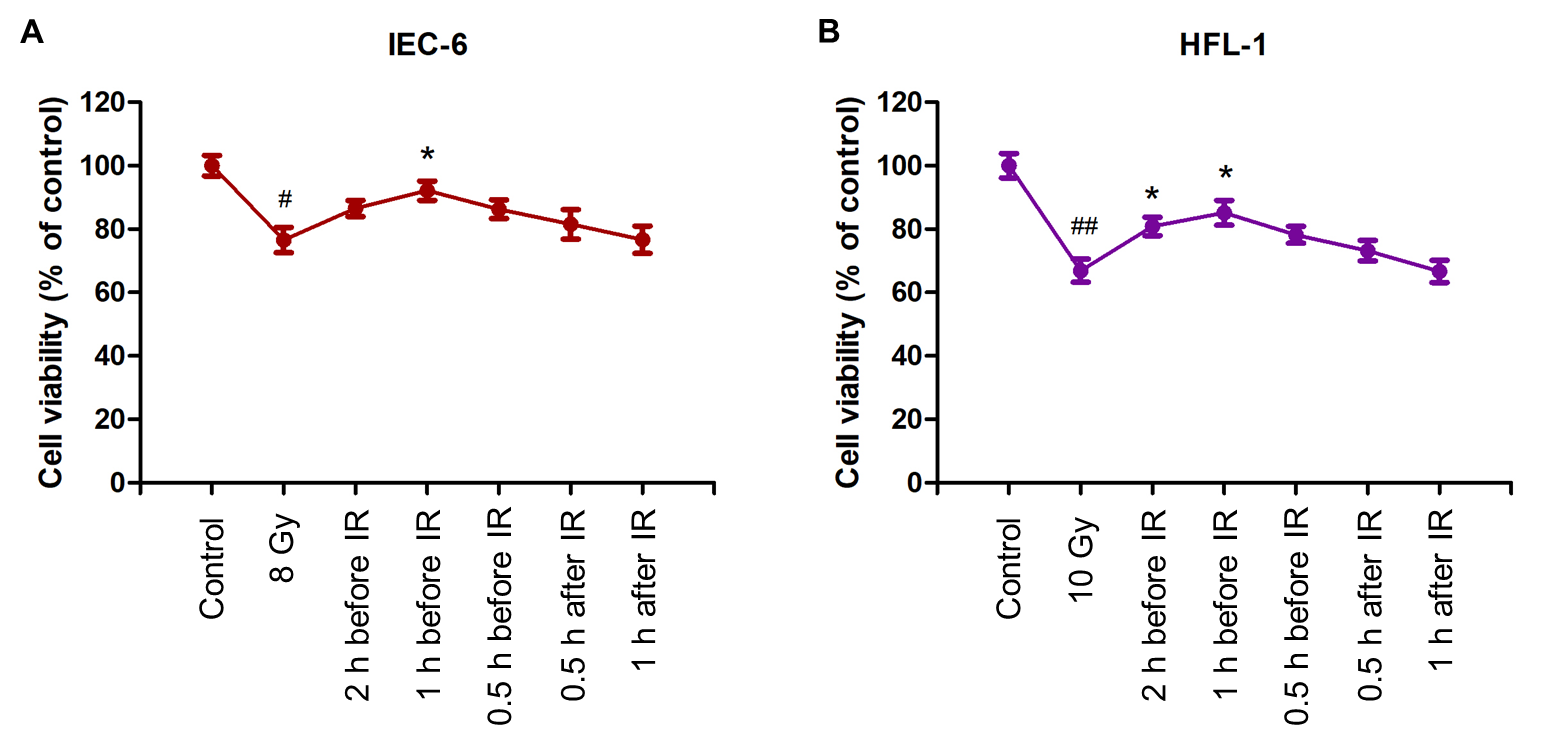
**

**Figure S1** Radioprotective activity of compound **10a** on (A) IEC-6 and (B) HFL-1 cells. Data are presented as the mean ± SEM (*n* = 3). ^#^*p* < 0.05 compared with control group, ^##^*p* < 0.01 compared with control group, **p* < 0.05 compared with the IR group.


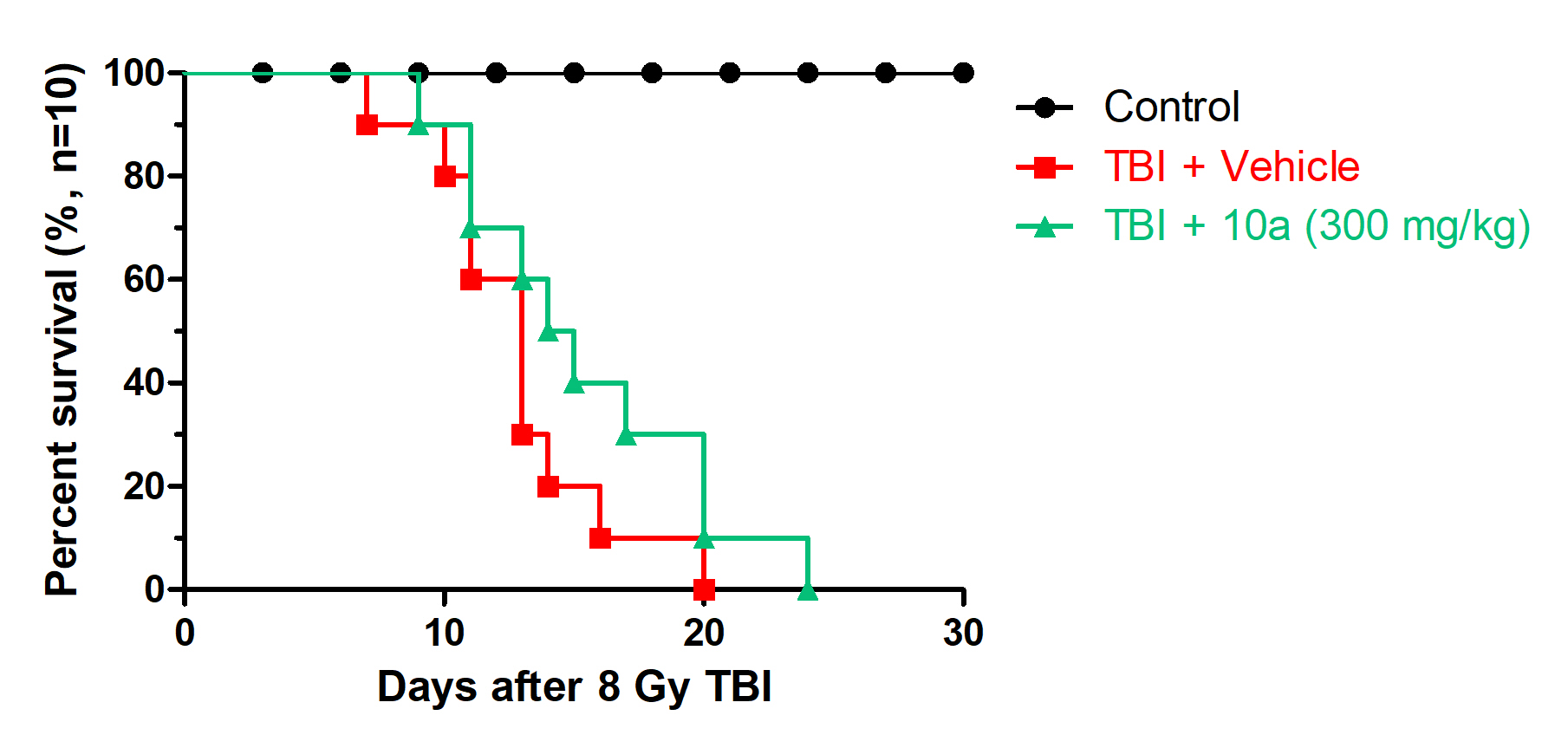


**Figure S2** Survival analysis of mice exposed to 8.0 Gy TBI.

**Analysis spectra for** **all intermediates, compound 9 and target compounds 10a–10f (Figure S3-S32)**


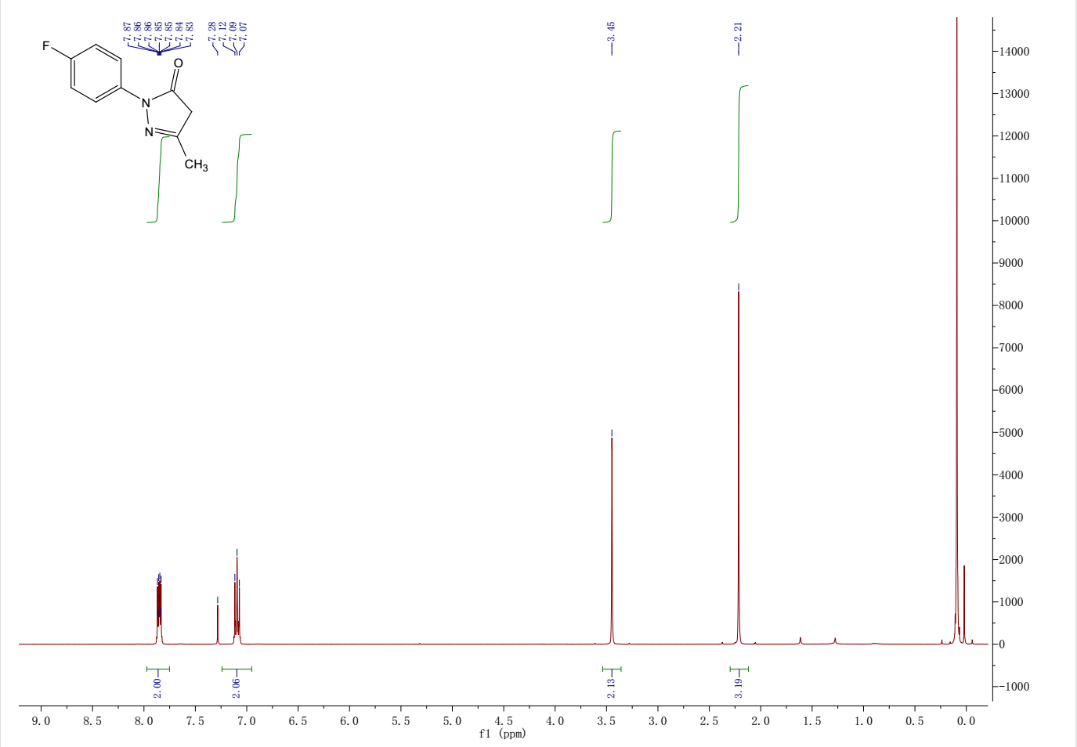


**Figure S3** ^1^H NMR spectrum of compound **2**.


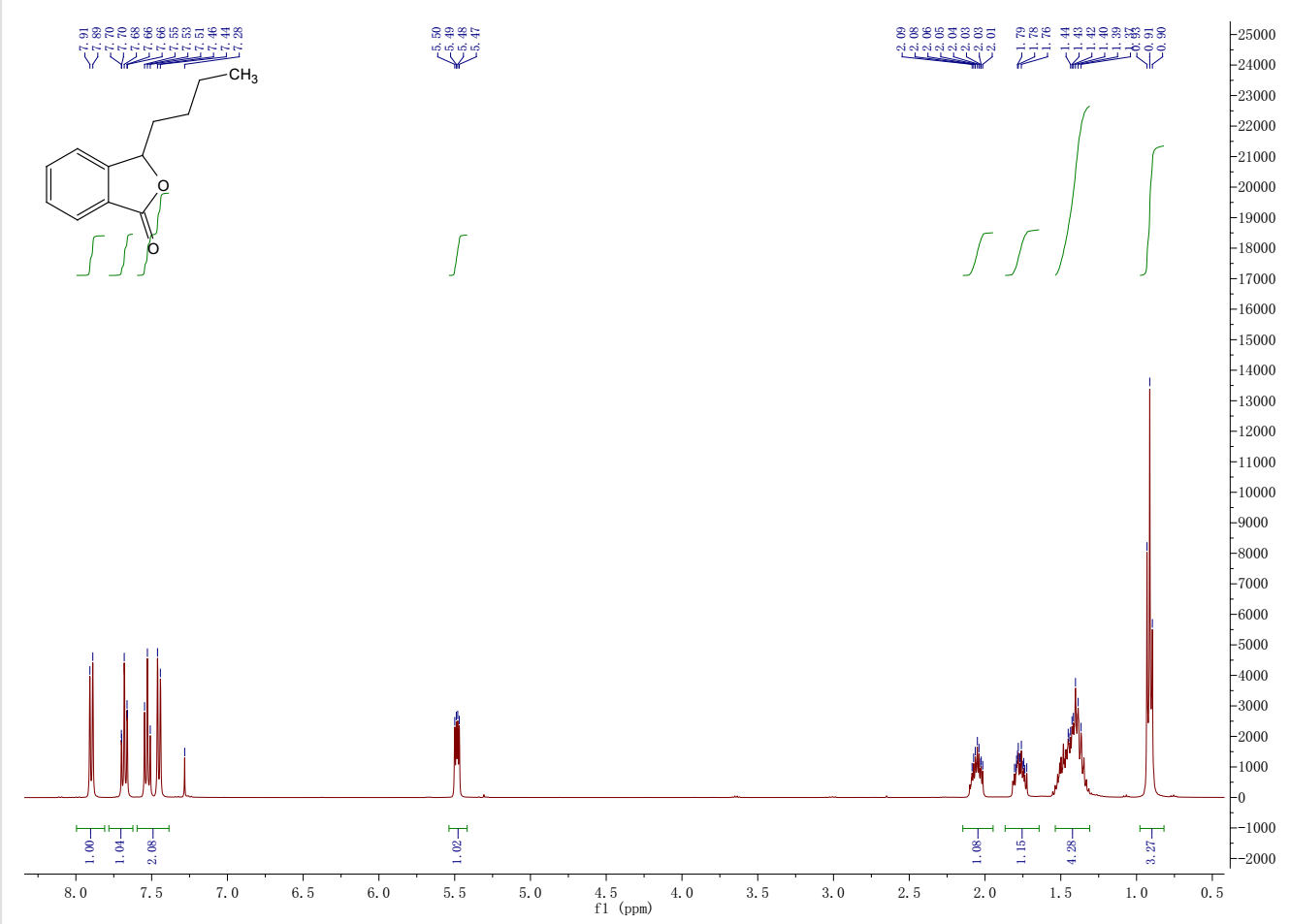


**Figure S4** ^1^H NMR spectrum of compound **4** (NBP).


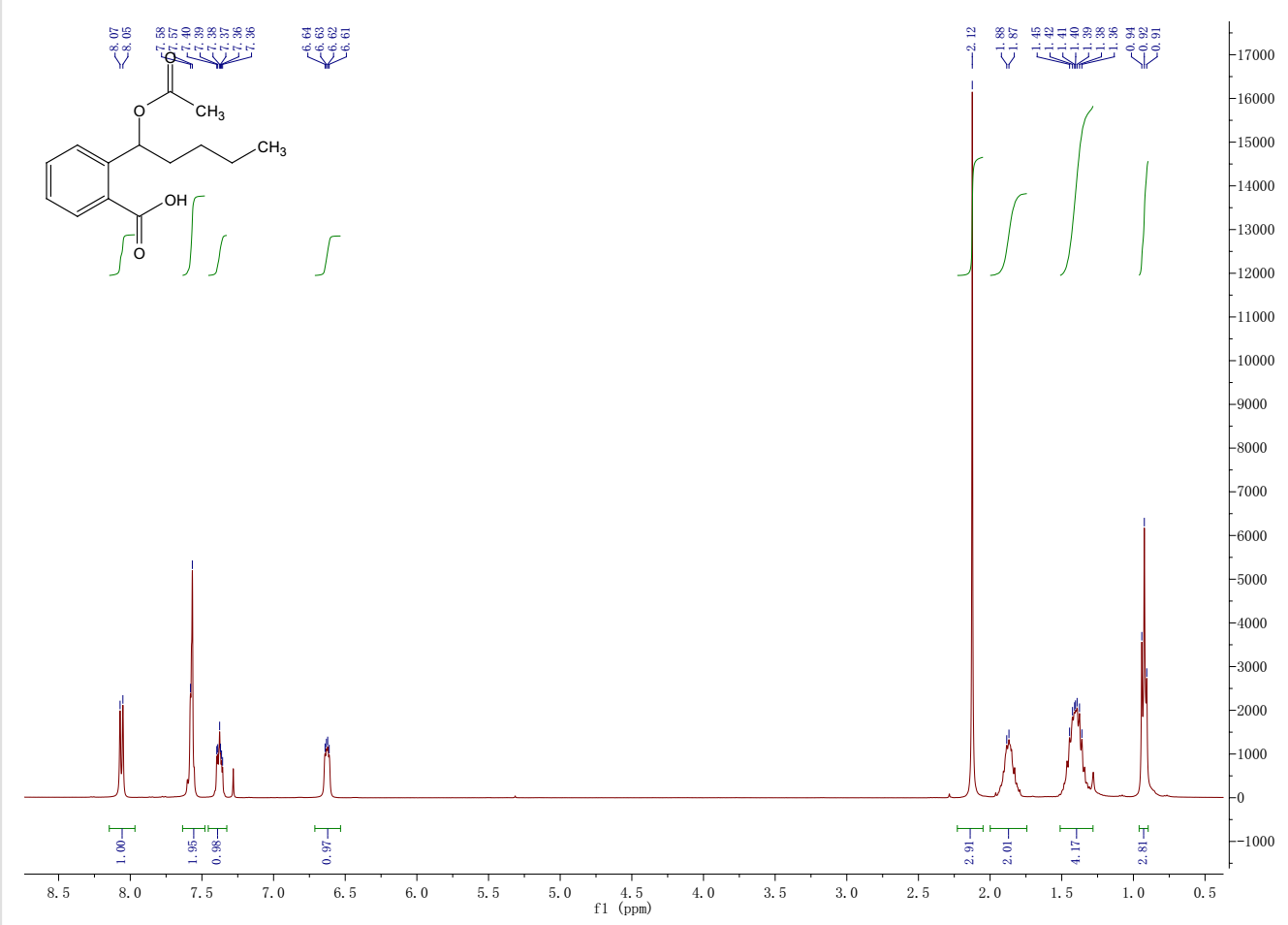


**Figure S5** ^1^H NMR spectrum of compound **6**.


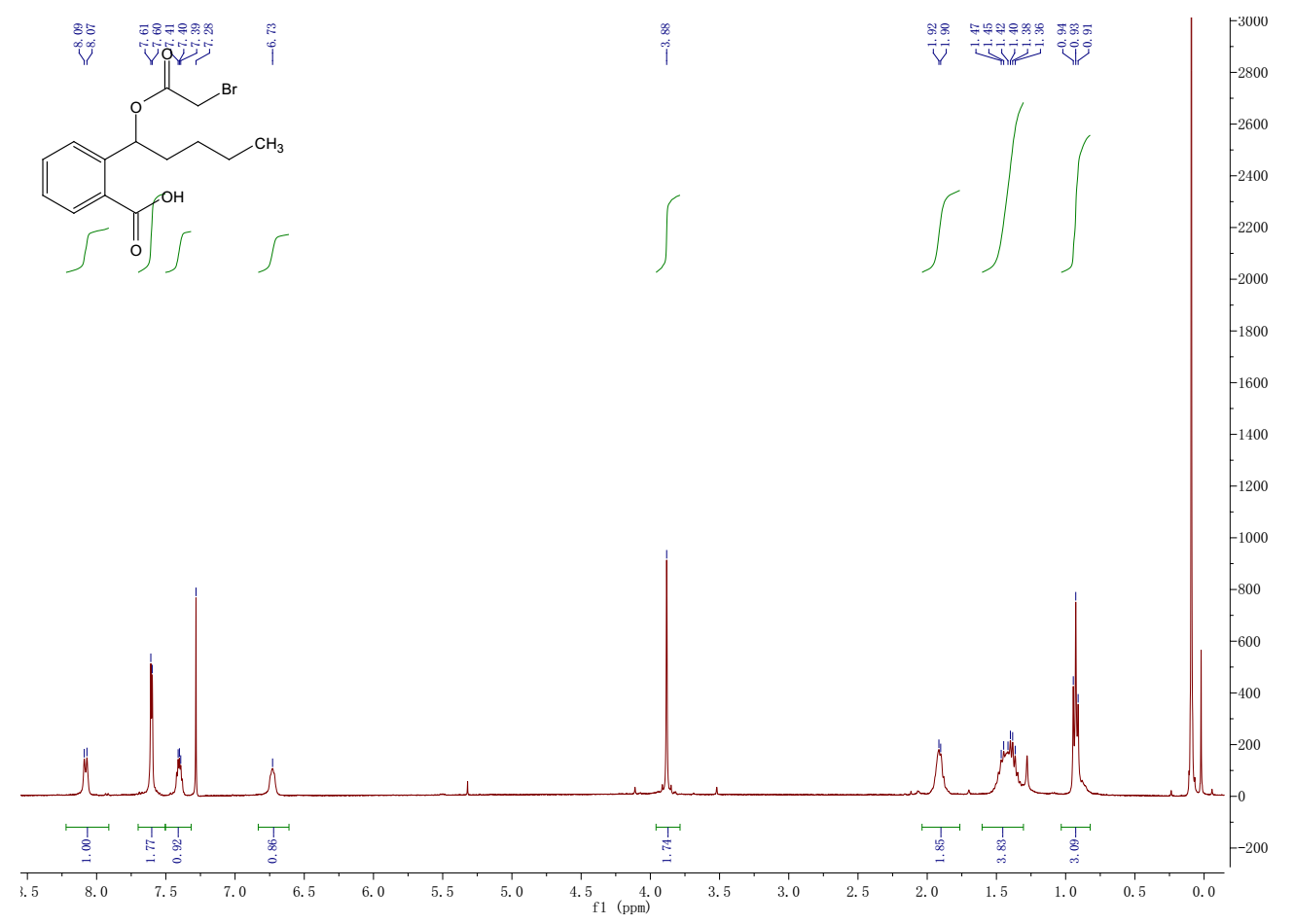


**Figure S6** ^1^H NMR spectrum of compound **7**.


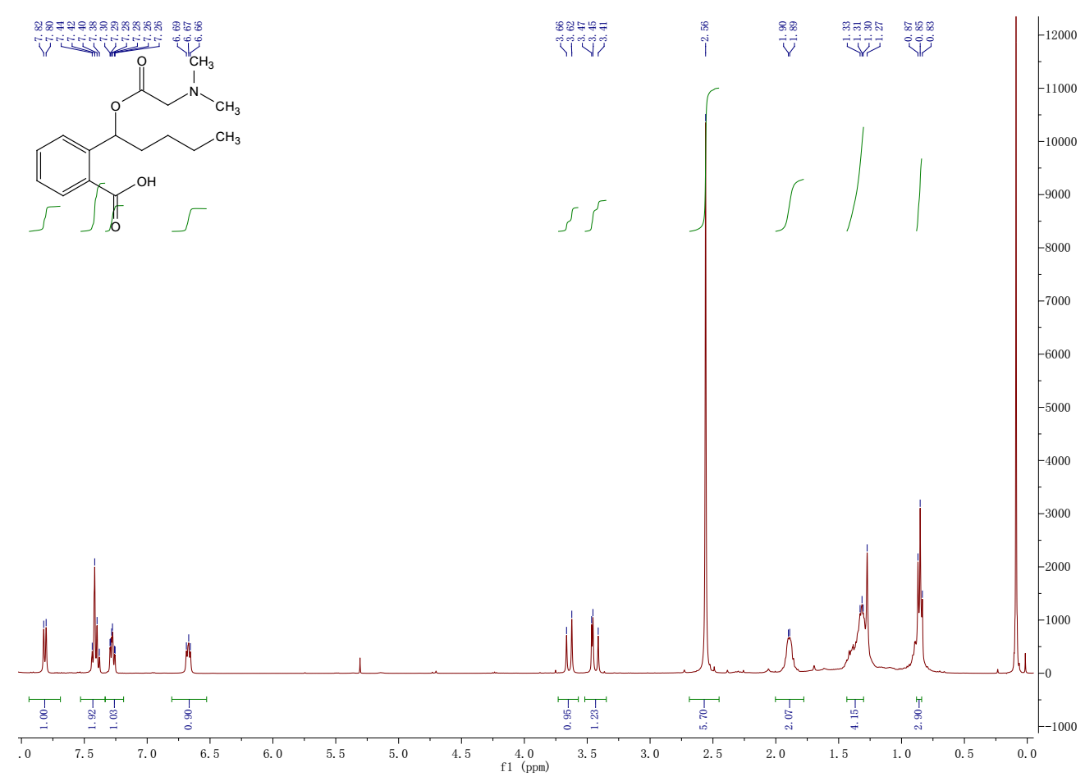


**Figure S7** ^1^H NMR spectrum of compound **8a**.


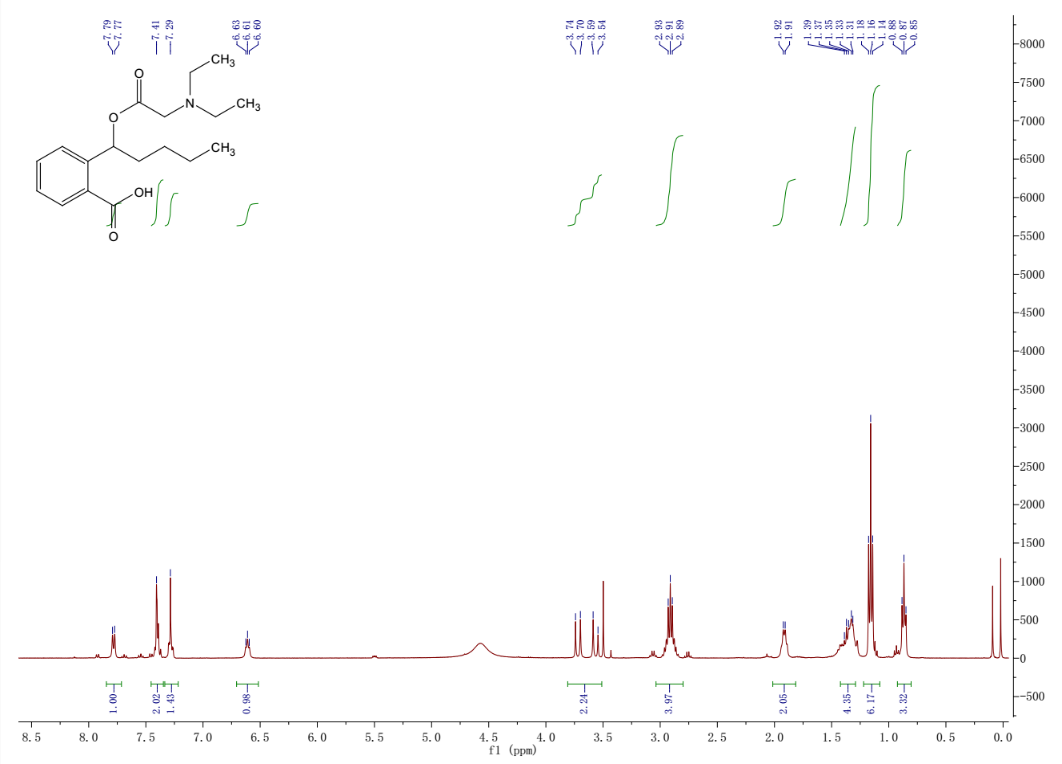


**Figure S8** ^1^H NMR spectrum of compound **8b**.


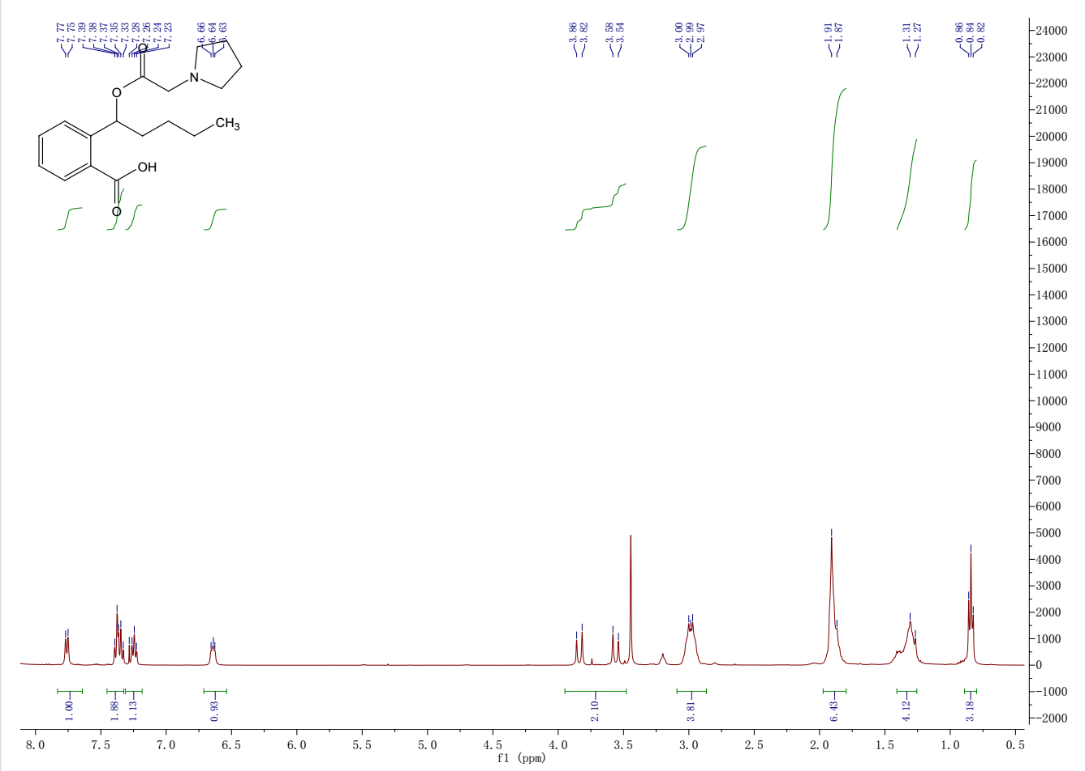


**Figure S9** ^1^H NMR spectrum of compound **8c**.


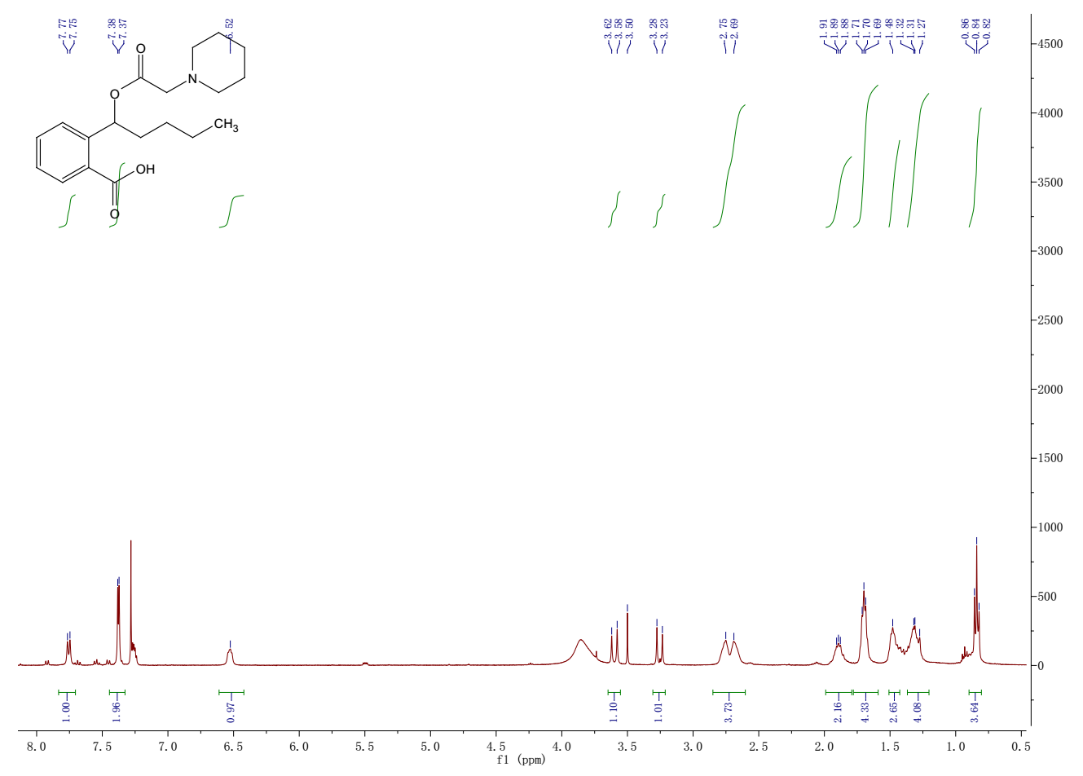


**Figure S10** ^1^H NMR spectrum of compound **8d**.


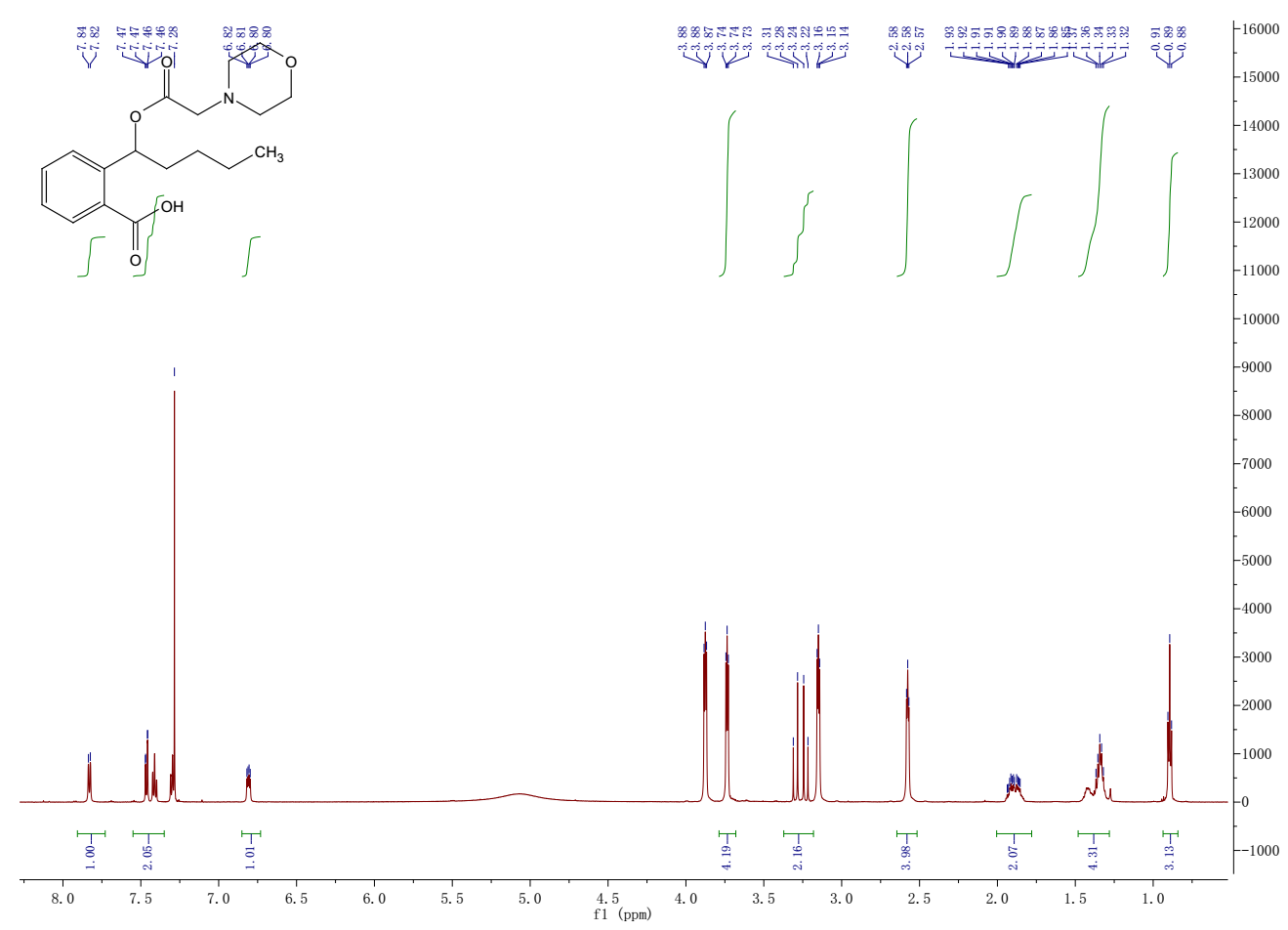


**Figure S11** ^1^H NMR spectrum of compound **8e**.


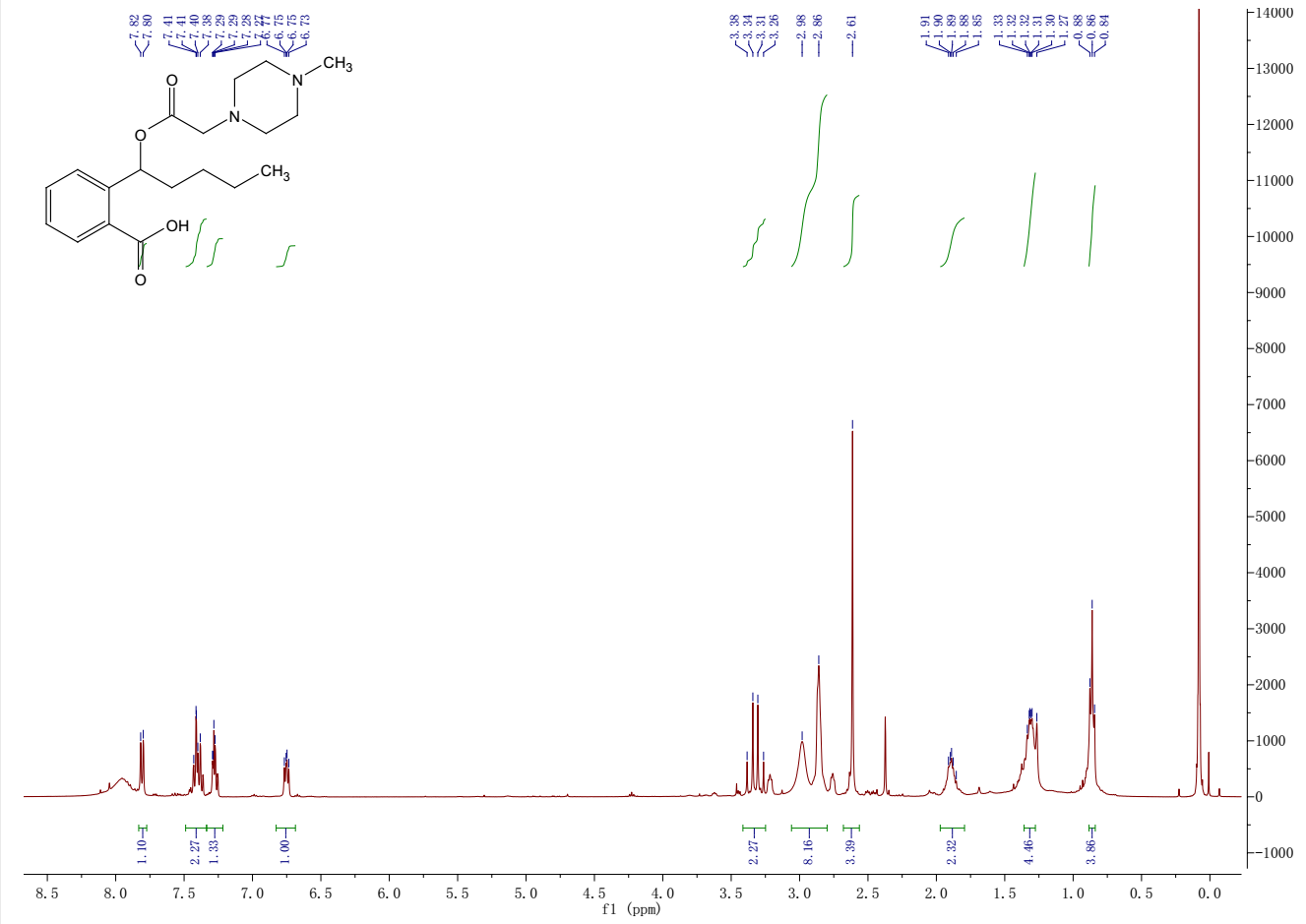


**Figure S12** ^1^H NMR spectrum of compound **8f**.


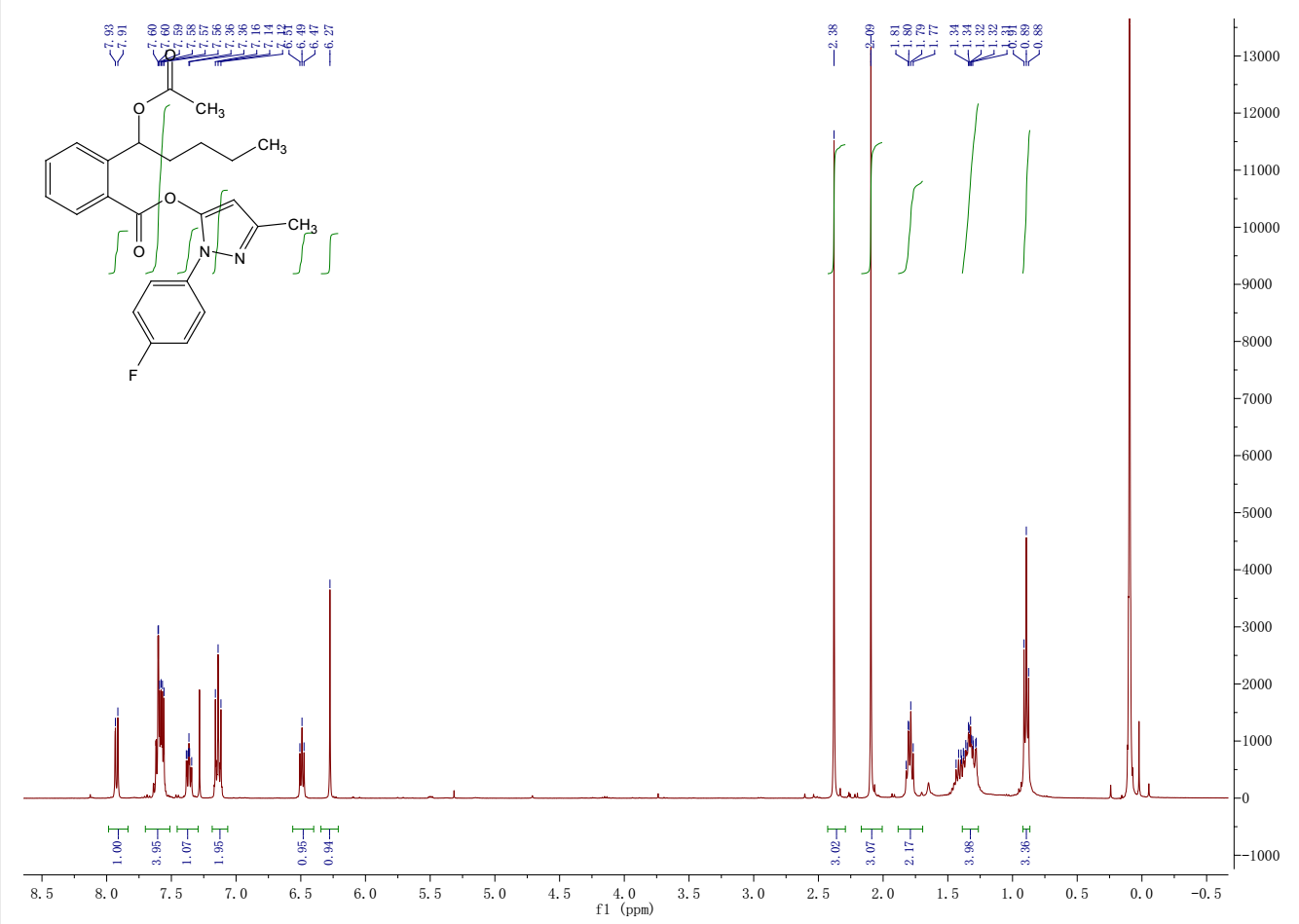


**Figure S13** ^1^H NMR spectrum of compound **9**.


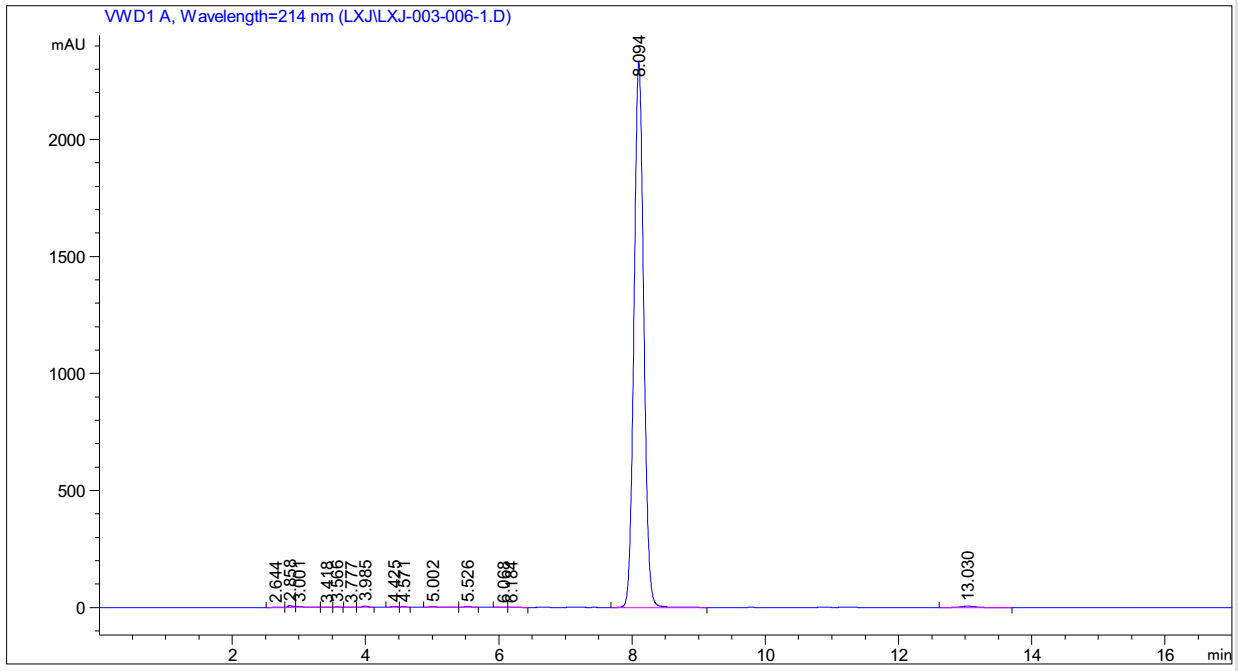


Wavelength = 214 nm

| Peak | RT (min) | Width  (min) | Area (mAU*s) | Height  (mAU) | Area (%) |
| --- | --- | --- | --- | --- | --- |
|  | 8.094 | 0.1578 | 2.37248e4 | 2330.21289 | 98.4983 |

**Figure S14** HPLC spectrum of compound **9**.


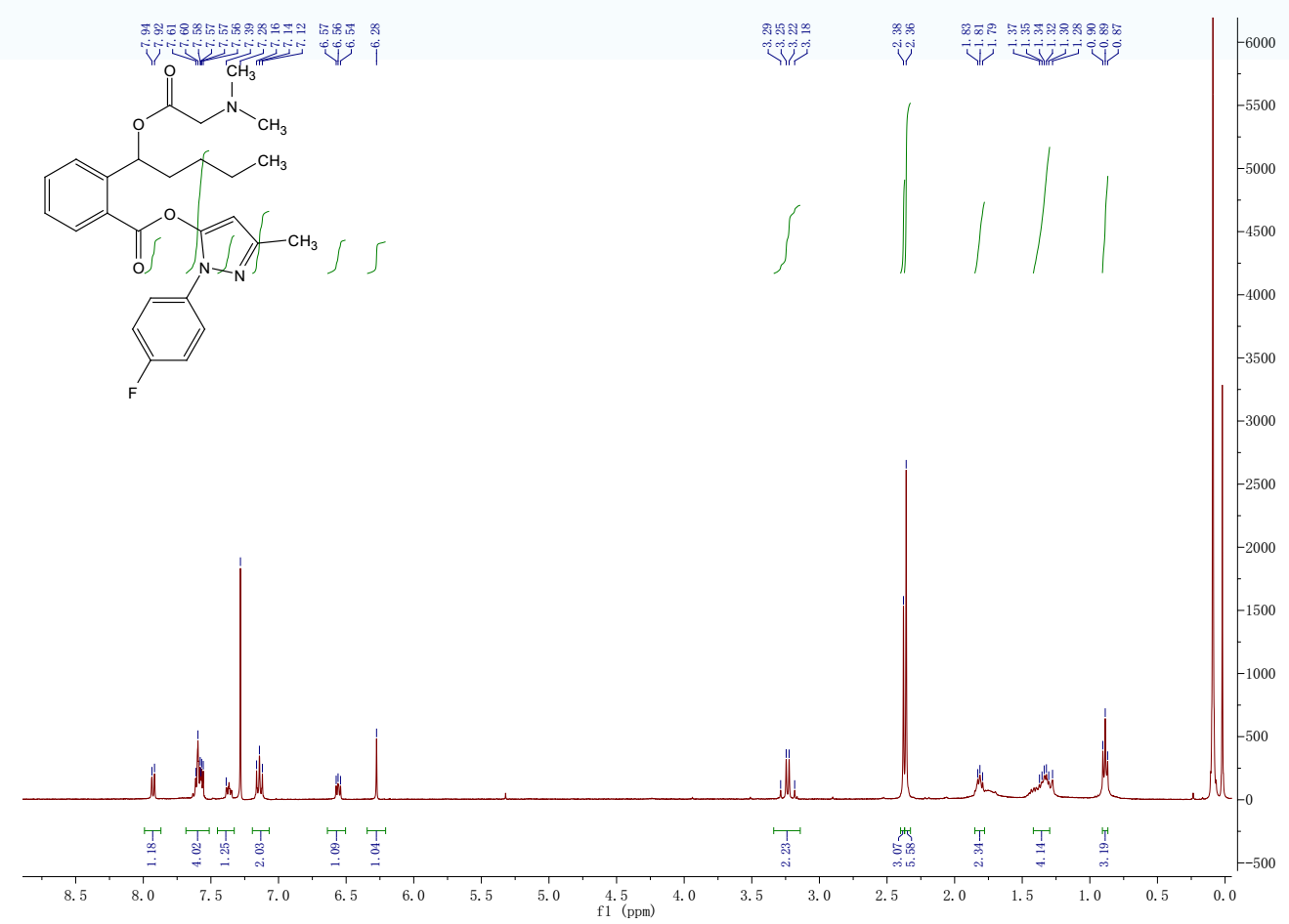


**Figure S15** ^1^H NMR spectrum of compound **10a**.


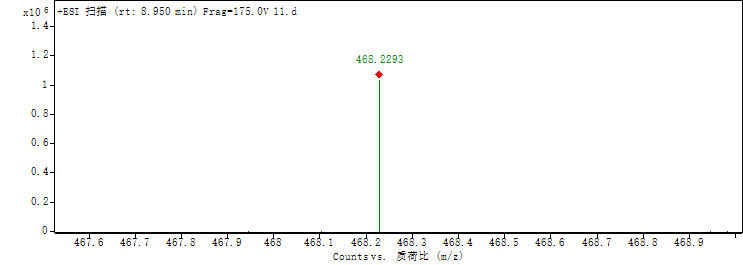


**Figure S16** HRMS spectrum of compound **10a**.


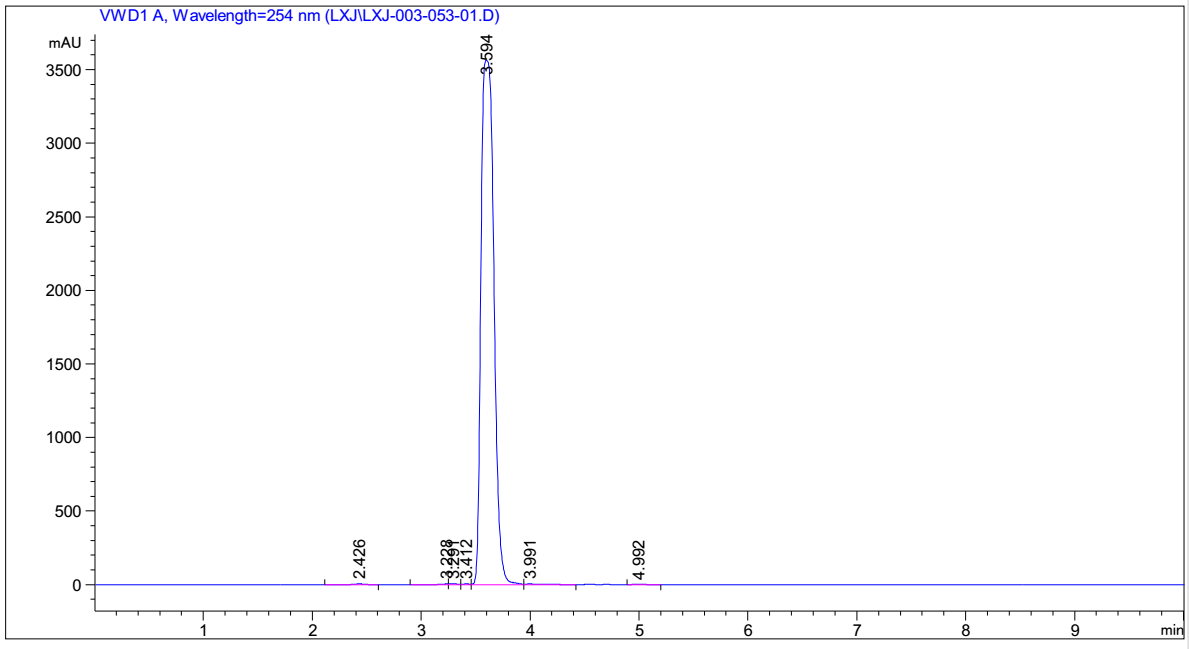


Wavelength = 254 nm

| Peak | RT (min) | Width  (min) | Area (mAU*s) | Height  (mAU) | Area (%) |
| --- | --- | --- | --- | --- | --- |
|  | 3.594 | 0.1316 | 2.92007e4 | 3563.28369 | 99.4603 |

**Figure S17** HPLC spectrum of compound **10a**.


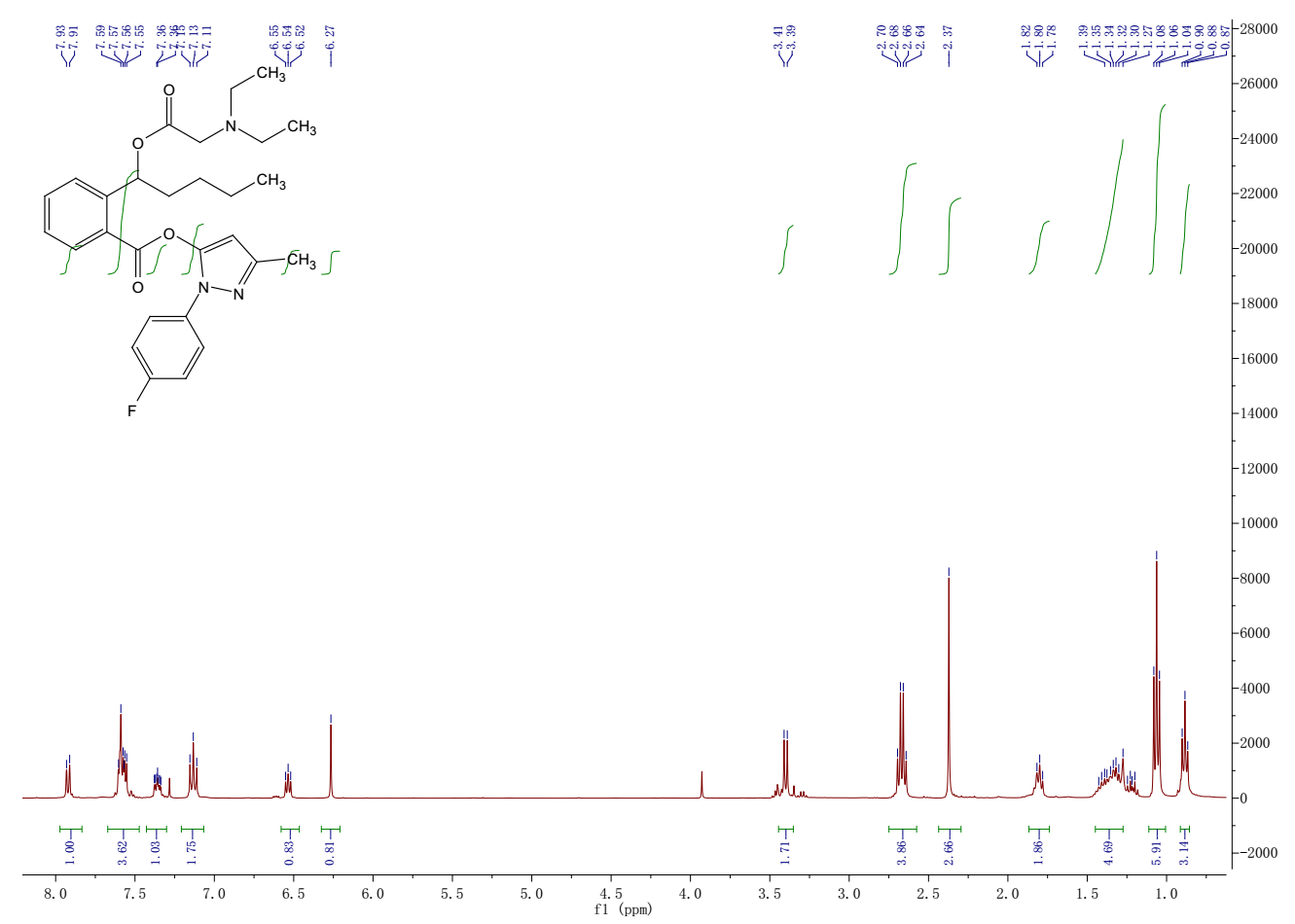


**Figure S18** ^1^H NMR spectrum of compound **10b**.


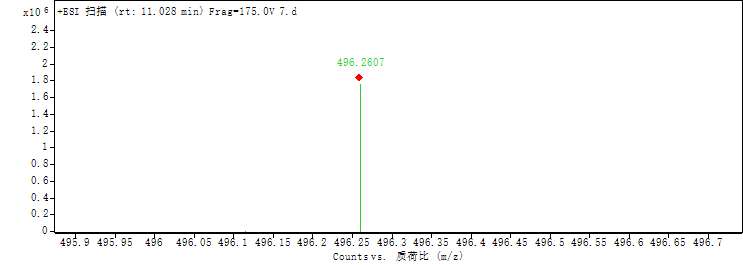


**Figure S19** HRMS spectrum of compound **10b**.


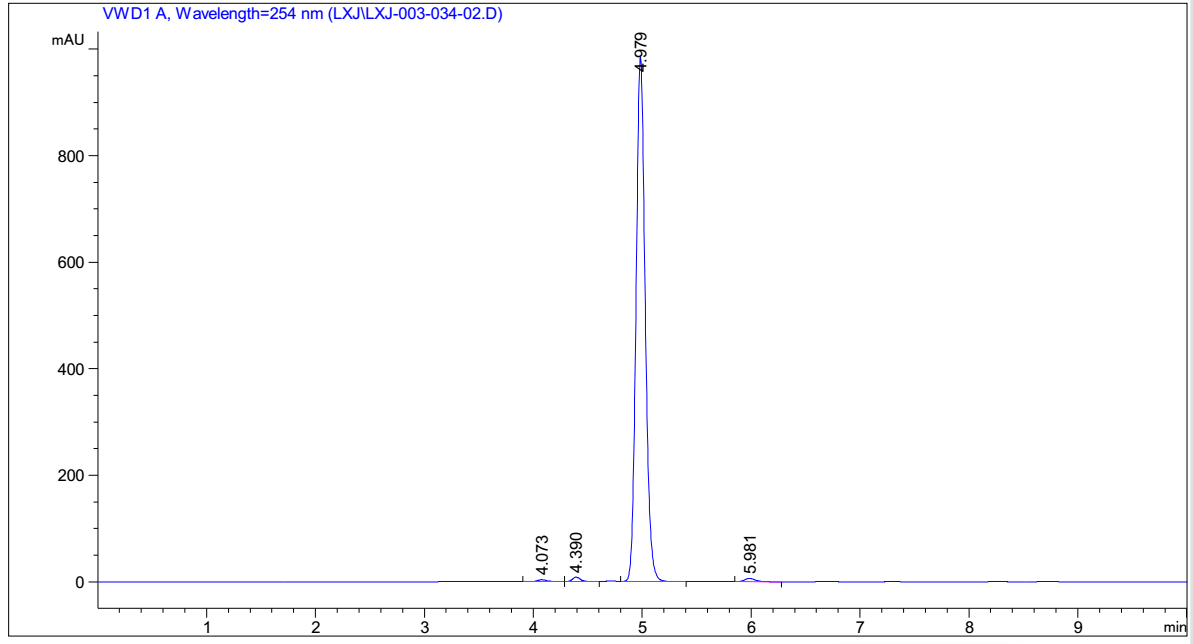


Wavelength = 254 nm

| Peak | RT (min) | Width  (min) | Area (mAU*s) | Height  (mAU) | Area (%) |
| --- | --- | --- | --- | --- | --- |
|  | 4.979 | 0.0900 | 5736.72412 | 984.23474 | 98.1136 |

**Figure S20** HPLC spectrum of compound **10b**.


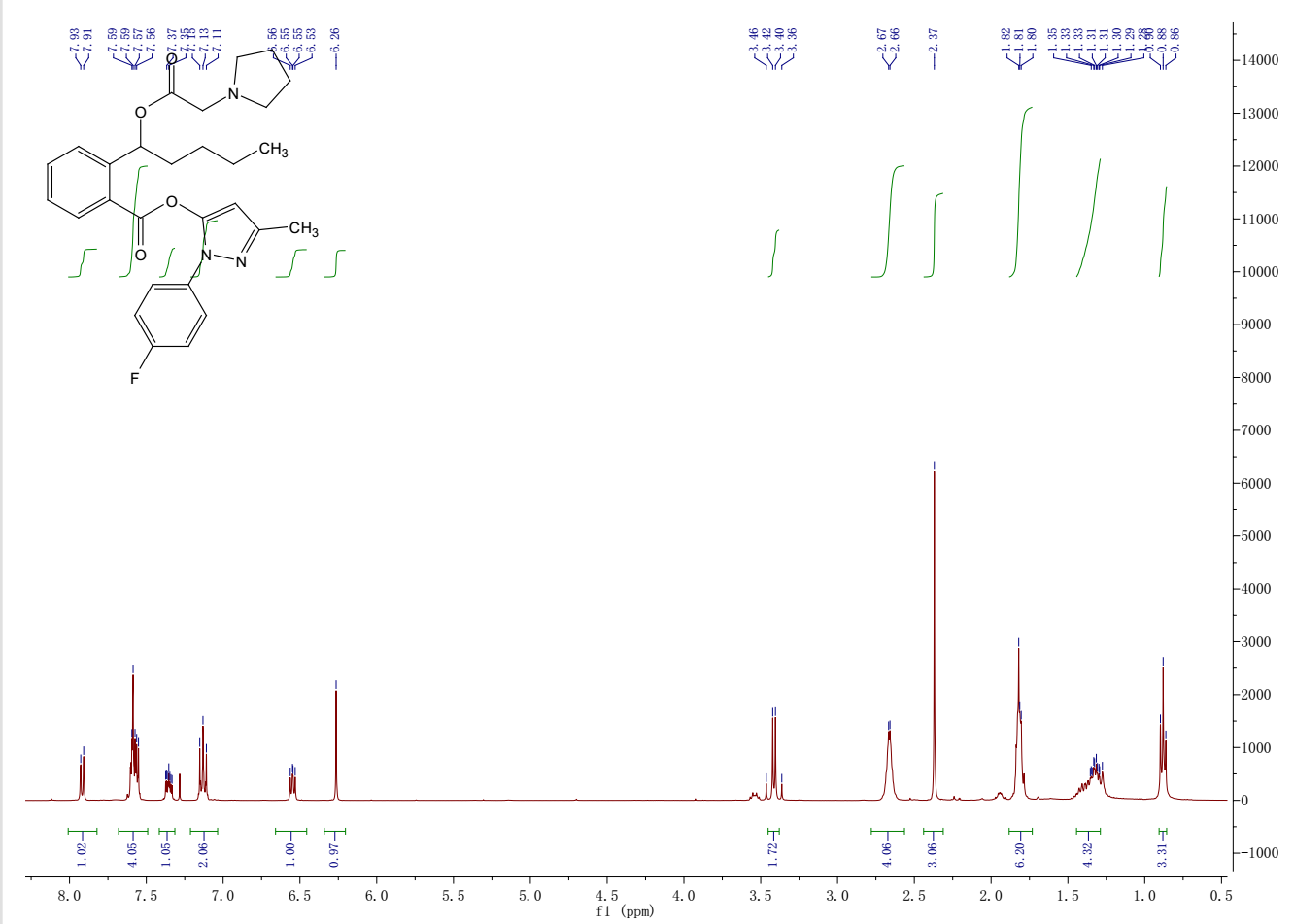


**Figure S21** ^1^H NMR spectrum of compound **10c**.


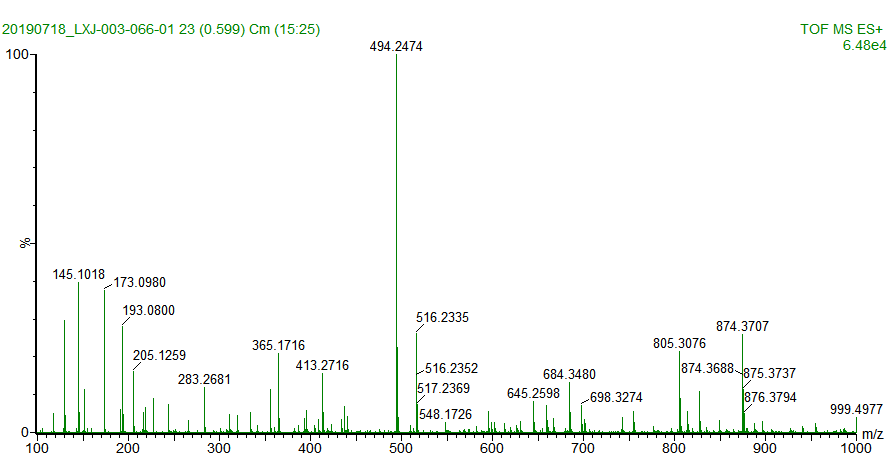


**Figure S22** HRMS spectrum of compound **10c**.


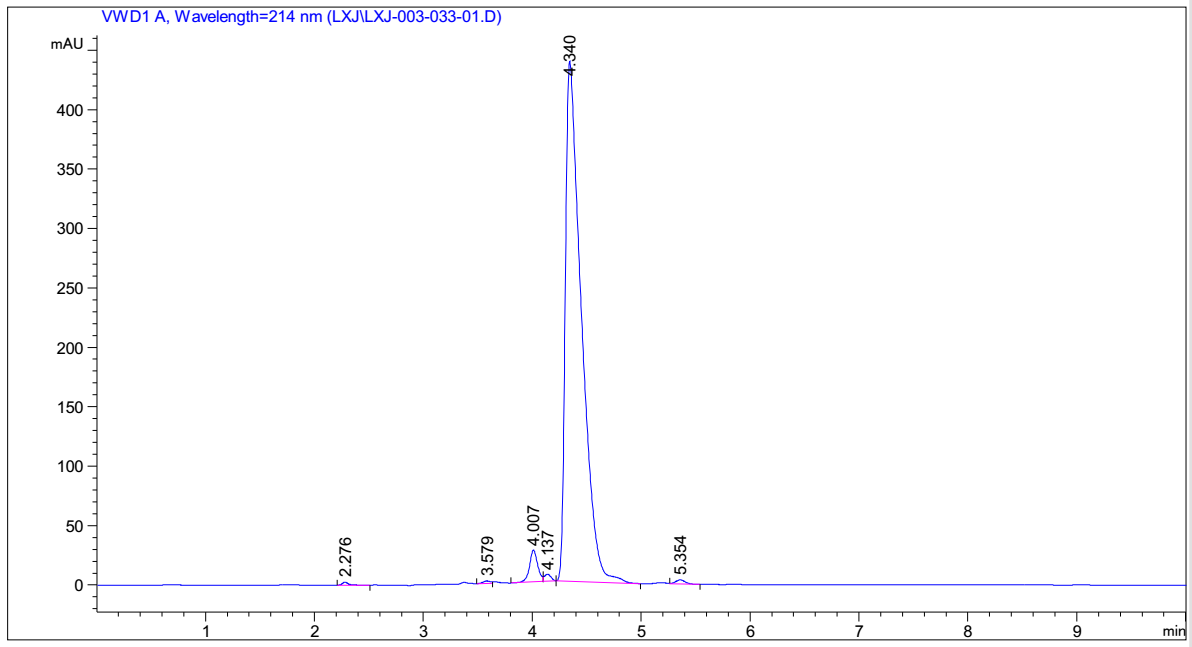


Wavelength = 214 nm

| Peak | RT (min) | Width  (min) | Area (mAU*s) | Height  (mAU) | Area (%) |
| --- | --- | --- | --- | --- | --- |
|  | 4.340 | 0.1459 | 4460.70459 | 437.60538 | 95.5579 |

**Figure S23** HPLC spectrum of compound **10c**.


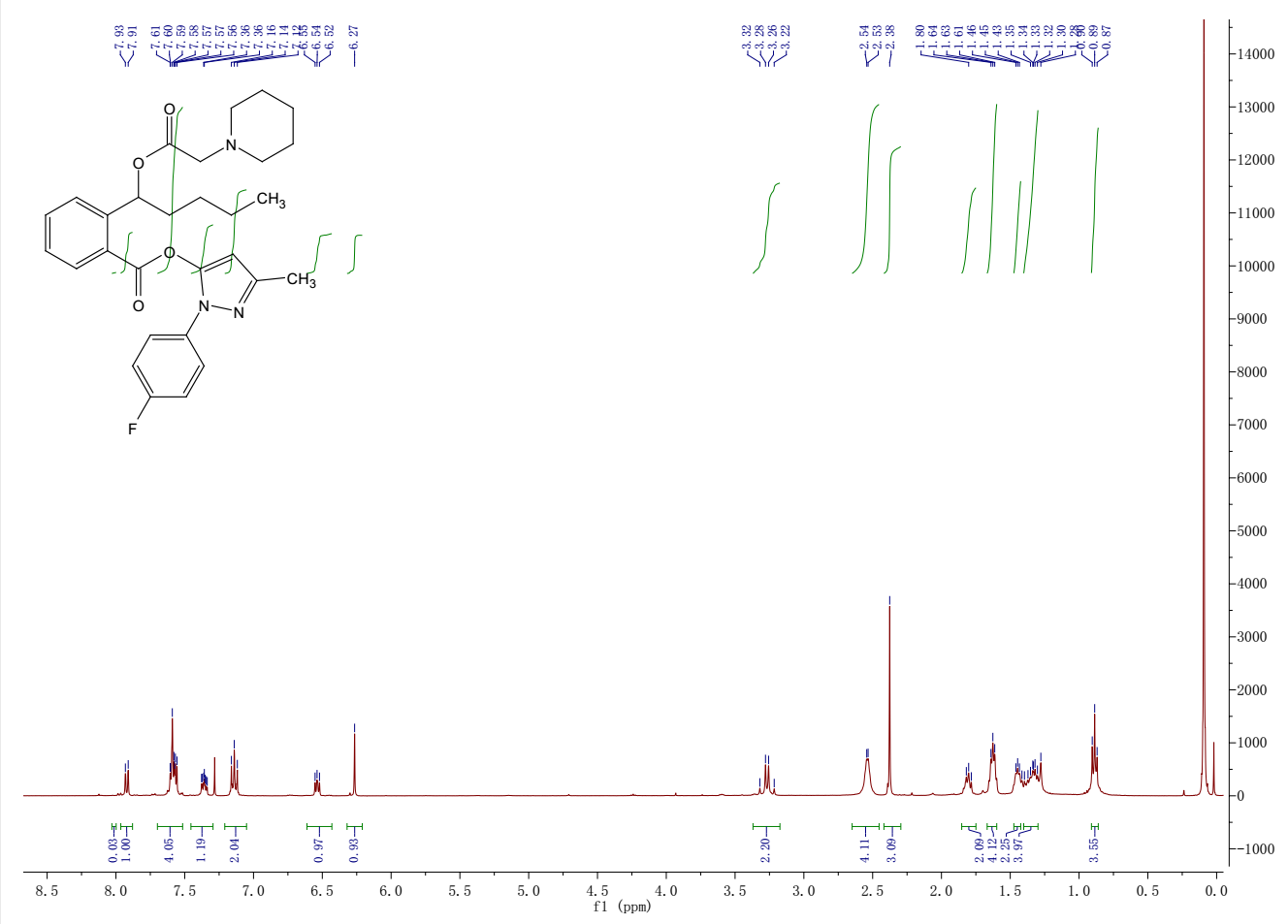


**Figure S24** ^1^H NMR spectrum of compound **10d**.


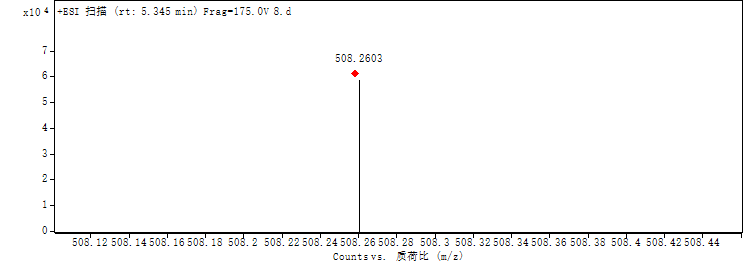


**Figure S25** HRMS spectrum of compound **10d**.


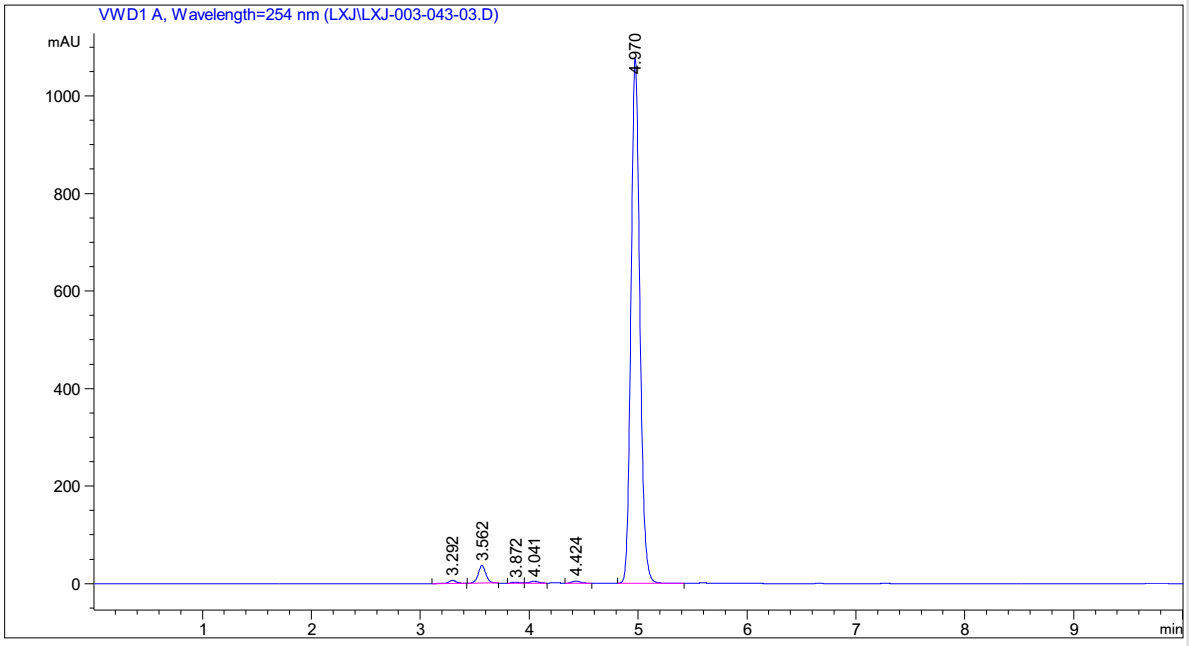


Wavelength = 254 nm

| Peak | RT (min) | Width  (min) | Area (mAU*s) | Height  (mAU) | Area (%) |
| --- | --- | --- | --- | --- | --- |
|  | 4.970 | 0.0870 | 6079.39551 | 1075.21033 | 95.8397 |

**Figure S26** HPLC spectrum of compound **10d**.


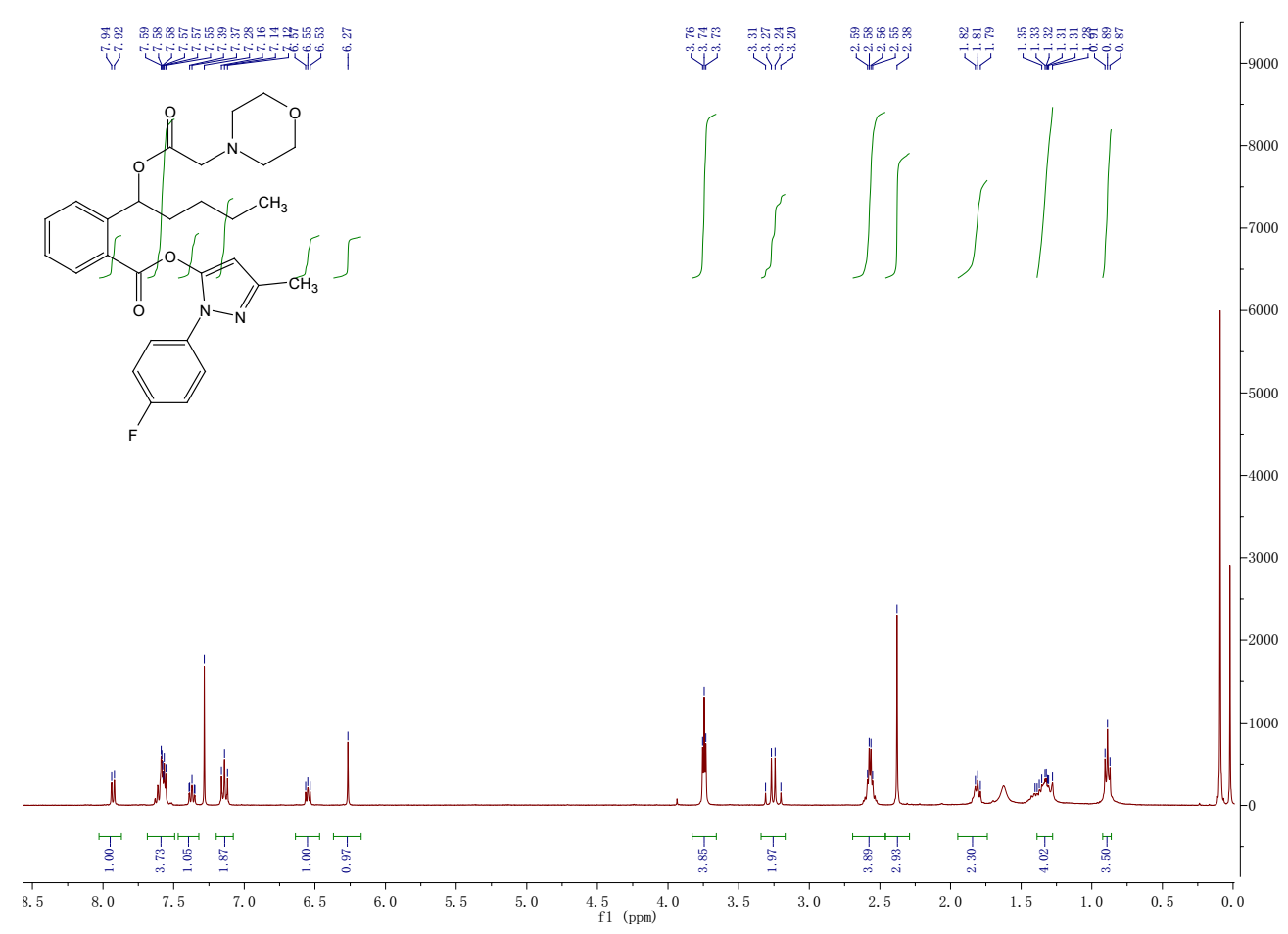


**Figure S27** ^1^H NMR spectrum of compound **10e**.


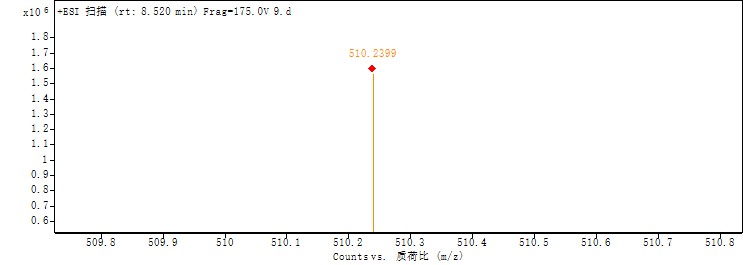


**Figure S28** HRMS spectrum of compound **10e**.


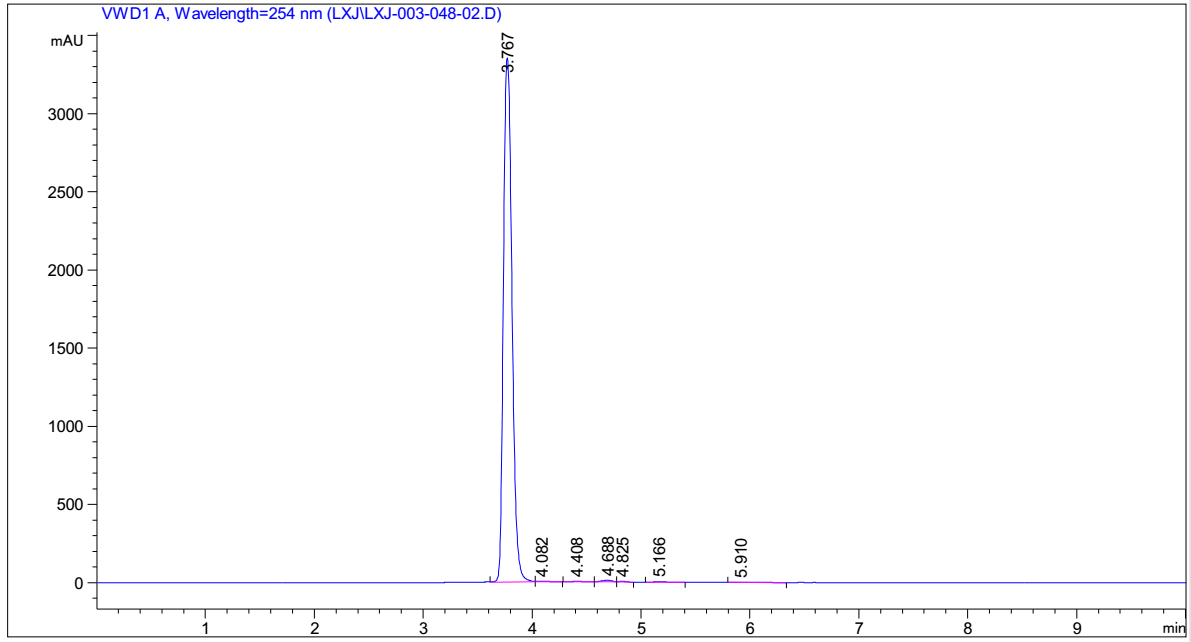


Wavelength = 254 nm

| Peak | RT (min) | Width  (min) | Area (mAU*s) | Height  (mAU) | Area (%) |
| --- | --- | --- | --- | --- | --- |
|  | 3.767 | 0.0868 | 1.83409e4 | 3351.66968 | 98.8548 |

**Figure S29** HPLC spectrum of compound **10e**.


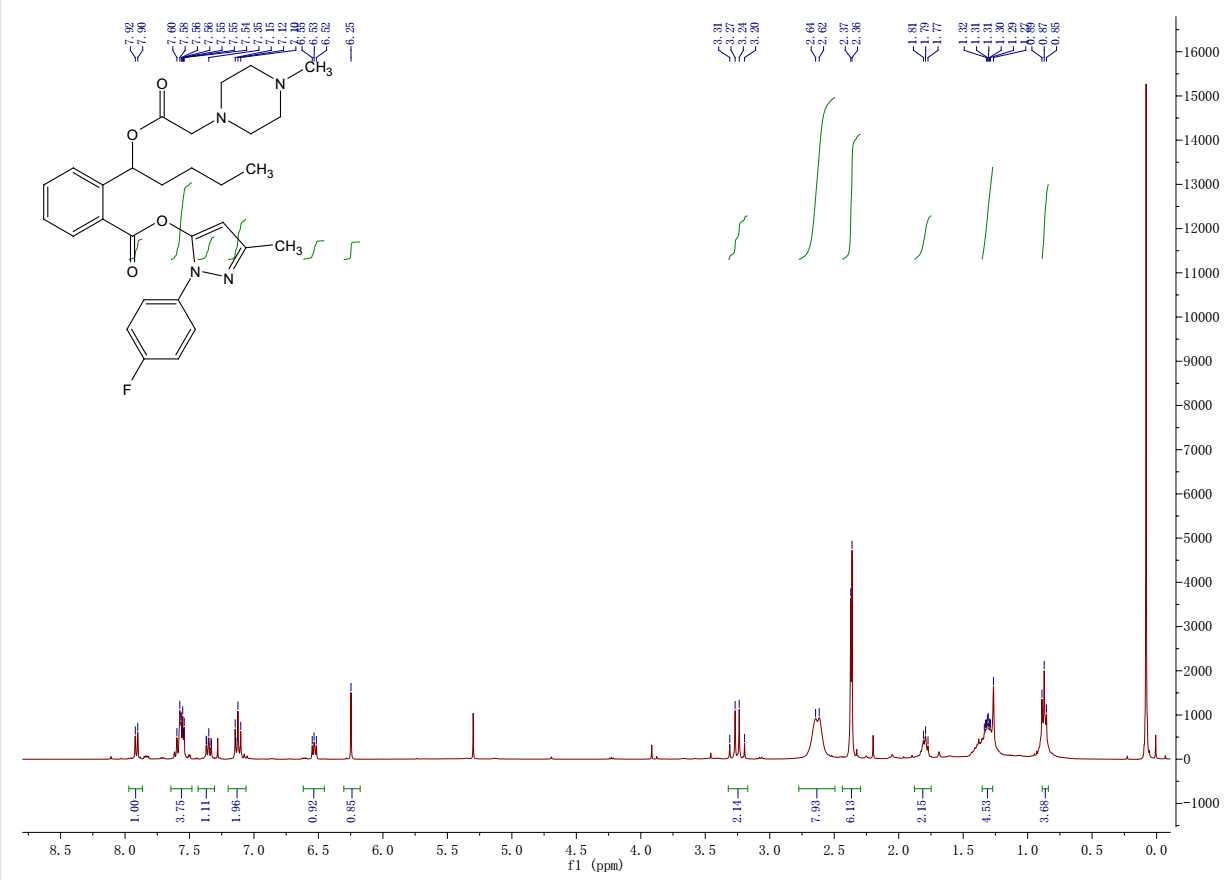


**Figure S30** ^1^H NMR spectrum of compound **10f**.

**Figure S31** HRMS spectrum of compound **10f**.


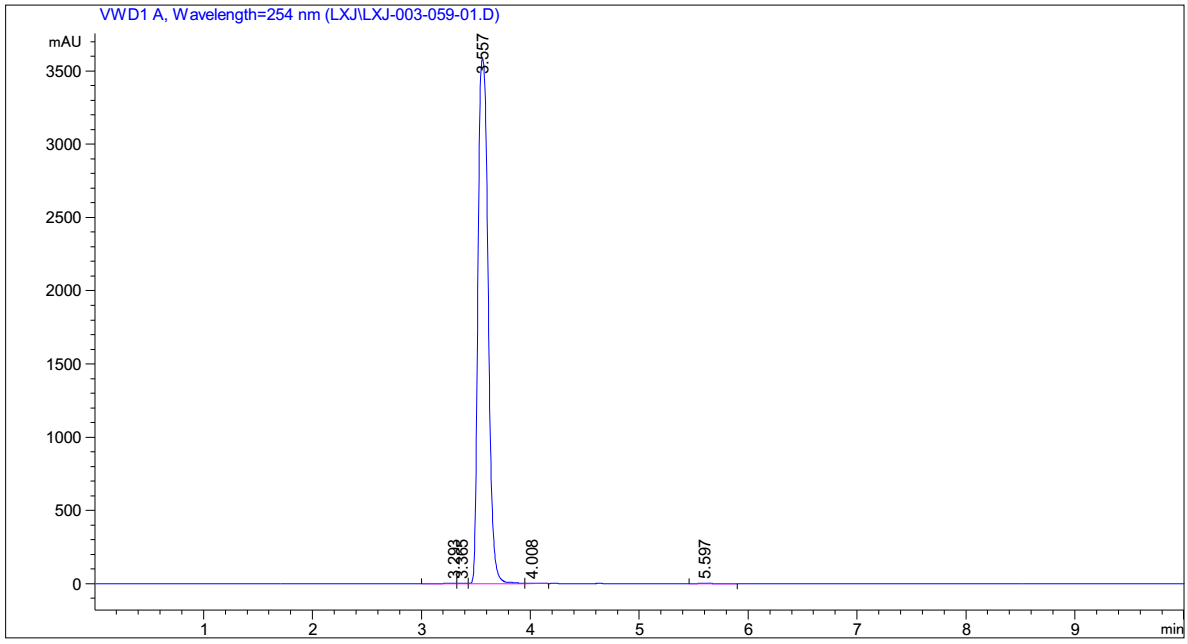


Wavelength = 254 nm

| Peak | RT (min) | Width  (min) | Area (mAU*s) | Height  (mAU) | Area (%) |
| --- | --- | --- | --- | --- | --- |
|  | 3.557 | 0.1031 | 2.30415e4 | 3579.38013 | 99.7568 |

**Figure S32** HPLC spectrum of compound **10f**.
